# Global burden of diphtheria, 1990–2021: a 204-country analysis of socioeconomic inequality based on SDI and DTP3 vaccination differences before and after the COVID-19 pandemic (GBD 2021)

**DOI:** 10.3389/fpubh.2025.1597076

**Published:** 2025-06-20

**Authors:** Zilun Wu, Liyu Lin, Jinbu Zhang, Jirui Zhong, Donglan Lai

**Affiliations:** ^1^The First Clinical Medical College, Guangzhou University of Chinese Medicine, Guangzhou, China; ^2^The First Affiliated Hospital of Guangzhou University of Chinese Medicine, Guangzhou, China; ^3^Guangdong Clinical Research, Academy of Chinese Medicine, Guangzhou, China

**Keywords:** diphtheria, Global Burden of Disease, socio-demographic index, vaccination, regional disparities, Joinpoint regression

## Abstract

**Objective:**

This study aimed to comprehensively assess the temporal trends and regional disparities in the global burden of diphtheria across 204 countries and territories from 1990 to 2021, as well as evaluate the impact of vaccination coverage on disease transmission.

**Methods:**

Data were extracted from the Global Burden of Disease (GBD) 2021 study. Statistical approaches, including Joinpoint regression, nonlinear fitting, and calculation of the average annual percentage change (AAPC), were employed to analyze diphtheria incidence, mortality, and disability-adjusted life years (DALYs). The Pearson correlation coefficient was used to quantify the association between diphtheria-tetanus-pertussis (DTP3) vaccination coverage and incidence. Analyses were stratified by region, sex, age, and socio-demographic index (SDI).

**Results:**

Between 1990 and 2021, the global age-standardized incidence rate (ASIR), mortality rate (ASMR), and DALY rate (ASMR) of diphtheria declined by 86.7, 75.0, and 86.4%, respectively, yet marked regional disparities persisted. Higher SDI levels correlated with reduced burden, with low-SDI regions accounting for 88.4% of global DALYs in 2021, while high-SDI regions approached elimination. DTP3 coverage negatively correlated with incidence (*r* = −0.191, *p* = 0.011), suggesting vaccination may reduce disease burden. We further analyzed confounders like SDI, drug resistance, health systems, surveillance gaps, and economic development. During the COVID-19 pandemic, global DTP3 coverage declined from 86 to 81%. Despite continued reductions in incidence—potentially linked to respiratory protective measures and historical immunization buffering—post-pandemic vaccination programs must be prioritized to mitigate resurgence risks, particularly in low-SDI and endemic regions.

**Conclusion:**

Over the past three decades, global efforts have significantly reduced the diphtheria burden, but systemic challenges remain in low-SDI regions. Vaccination remains pivotal; however, post-pandemic declines in coverage underscore latent epidemic risks. Low-SDI regions require integrated nutrition-vaccination strategies, conflict-adapted delivery, and robust cold chains, while high-SDI areas should enhance genomic surveillance, adult boosters, and equitable technology sharing. This study provides the first systematic quantification of spatiotemporal diphtheria burden patterns, offering critical evidence for the WHO 2030 Diphtheria Elimination Roadmap. Eliminating regional disparities necessitates strengthened healthcare systems and adult booster immunization strategies.

## 1 Introduction

Diphtheria, an acute respiratory infectious disease caused by *Corynebacterium diphtheriae*, remains a persistent threat to global public health due to its severe complications and high mortality rates (1). Vaccination represents one of the most successful and cost-effective public health interventions of the last century, having saved millions of lives (2). Although the widespread administration of diphtheria-containing vaccines has significantly reduced the disease burden, regional disparities persist, particularly in resource-limited settings where insufficient immunization coverage, fragile healthcare systems, and lagging socio-demographic development contribute to ongoing disease transmission. According to the World Health Organization (WHO), an estimated 13,000 new cases were reported globally in 2021, with 62% of disability-adjusted life years (DALYs) concentrated in sub-Saharan Africa.

The COVID-19 pandemic led to a global shift in vaccination priorities, resulting in decreased routine immunization coverage, particularly among children, as reported by WHO and UNICEF in September 2024. This decline has heightened the resurgence risk of vaccine-preventable diseases (e.g., measles, pneumonia), underscoring the urgent need for targeted strategies in low-coverage regions to address both gaps in delivery and vaccine hesitancy. Enhanced monitoring and data systems are critical to improving coverage estimates and guiding policy decisions (3). While some countries have implemented catch-up campaigns post-pandemic (4), disparities in childhood vaccine access have worsened, especially in low-and middle-income countries (LMICs), necessitating equitable sharing of vaccine R&D capacities (5). Regional outbreaks, such as the diphtheria resurgence in Nigeria and Haiti, highlight the imperative for context-specific control strategies (6), underscoring the urgent need for region-specific control strategies.

Previous studies have predominantly focused on the epidemiological history of diphtheria or vaccine effectiveness in individual countries, yet comprehensive analyses of long-term global trends, socio-demographic index (SDI)-driven health inequalities, and the dynamic relationship between vaccine coverage and disease incidence remain limited (7). Furthermore, the impact of the COVID-19 pandemic on routine immunization programs, including its lag effects and the subsequent decline in vaccination rates (8), has not been thoroughly evaluated. While the Global Burden of Disease (GBD) study provides cross-disease data, in-depth analyses of regional heterogeneity, age-specific disease burden, and social determinants specific to diphtheria remain insufficient.

Antimicrobial resistance (AMR) patterns of *Corynebacterium diphtheriae* have exhibited shifts following the pandemic, with marked disparities observed between high-and low-SDI (socio-demographic Index) countries. For instance, a study in South Africa (9) reported a high prevalence of intermediate penicillin resistance (82/84 [98%] isolates), while erythromycin susceptibility remained conserved (83/84 [99%] isolates). In contrast, the United Kingdom (10) documented an imported *C. diphtheriae* strain, with local antimicrobial susceptibility testing identifying six macrolide-resistant isolates (6/73). The emergence of macrolide-resistant *Bordetella pertussis* in Europe further raises concerns, prompting critical reflections on antibiotic usage. These findings suggest that the evolution of *C. diphtheriae* AMR is likely influenced by a complex interplay of geographic, ethnic, strain-specific, and epidemiological factors.

This study, based on GBD 2021 data, systematically evaluates the global epidemiological trends of diphtheria from 1990 to 2021, aiming to: (1) quantify spatiotemporal variations in incidence, mortality, and DALYs; (2) elucidate regional disparities under SDI stratification and the influence of socio-economic development on disease burden; (3) analyze the association between DTP3 (diphtheria-tetanus-pertussis) vaccine coverage and disease incidence, exploring optimized vaccination strategies; and (4) assess the short-term and potential long-term effects of the COVID-19 pandemic on immunization coverage and disease transmission.

Our findings provide robust empirical evidence to guide optimal resource allocation and immunization strategy development, directly contributing to WHO’s Immunization Agenda 2030 (IA2030) (11), and global diphtheria elimination efforts. Our research delivers actionable policy recommendations that consider regional healthcare disparities, life-course vaccination requirements, and local implementation capacities. By establishing evidence-based vaccination coverage targets (≥95%) and incorporating strain-specific risk evaluations, this work offers both a technical framework for national immunization programs and a strategic pathway toward global diphtheria eradication, while addressing current limitations in burden estimation and prevention prioritization.

The findings highlight that, although the global burden of diphtheria has significantly declined over the past three decades, certain countries continue to experience high incidence rates and disease burdens. Coupled with the persistent decline in vaccination rates following the COVID-19 pandemic, there is a potential risk of diphtheria resurgence. In low-SDI regions, priority should be given to strengthening childhood immunization and adult booster vaccination, while high-SDI regions must ensure sustained vaccination coverage and enhance active surveillance. Additionally, international collaboration is essential to improve vaccine accessibility, thereby accelerating progress toward the global control and elimination of diphtheria.

## 2 Materials and methods

### 2.1 Data sources

The 2021 Global Burden of Disease (GBD) study synthesizes epidemiological data from 204 countries and territories to assess the burden of diphtheria. This comprehensive dataset employs standardized methods to quantify health losses associated with 369 diseases, injuries, and impairments (12), as well as 88 risk factors, ensuring cross-regional comparability.

The GBD framework employs advanced statistical techniques to address missing data and reporting inconsistencies, particularly in conflict-affected and surveillance-limited regions (13, 14). It synthesizes traditional data (vital registration systems, surveillance reports, national surveys) with non-traditional sources (NGO reports, conflict databases, mobile health surveys) to overcome monitoring gaps. The framework applies Cause of Death Ensemble modeling (CODEm) and Bayesian meta-regression (DisMod-MR 2.1) for data harmonization, while spatiotemporal Gaussian process regression leverages data from neighboring regions to estimate burden in data-sparse areas. Population surveys and satellite data supplement conflict zone demographics. Through 1,000 bootstrap iterations, the model generates uncertainty intervals (UIs) to quantify estimation precision, with cross-validation ensuring robustness in low-data environments (15). This comprehensive approach systematically adjusts for demographic biases and reporting variations, providing reliable burden estimates where health infrastructure is weakest. All analyses adhere to the Guidelines for Accurate and Transparent Health Estimates Reporting (GATHER) (16).

Vaccination coverage data were obtained from the World Health Organization (WHO) Immunization Database (17), which provides country-level estimates of diphtheria-tetanus-pertussis (DTP3) vaccine coverage (18). These data, derived from administrative records and immunization surveys, were integrated into the analysis to assess the relationship between vaccination rates and diphtheria incidence. The WHO database also includes supplementary information on immunization program performance, such as dropout rates (19) and timeliness of vaccine delivery, which were used to contextualize regional disparities in disease burden. Ethical approval was waived by the Institutional Review Board of the First Affiliated Hospital of Guangzhou University of Chinese Medicine, as the study utilized anonymized, publicly available data.

### 2.2 Socio-demographic index

The socio-demographic index (SDI) quantifies national development (0–1 scale) using three components: lag-distributed income, educational attainment (age ≥ 15), and total fertility rate (20). Countries were classified into quintiles (low-high SDI) to analyze diphtheria burden disparities, as this approach: (1) enables cross-country comparisons, (2) captures epidemiological transition patterns, and (3) maintains GBD methodological consistency (21). Smooth spline models assessed nonlinear relationships between SDI and age-standardized incidence/mortality/DALY rates across 21 regions. The quintile stratification reflects meaningful development thresholds while ensuring sufficient sample sizes for robust analysis. This standardized classification system allows for effective comparison of disease burden across different socioeconomic contexts, with detailed methodology available in prior GBD publications (20).

### 2.3 DisMod-MR 2.1

DisMod-MR 2.1 (22), the Bayesian meta-regression tool developed by IHME for GBD studies, employs a compartmental modeling framework to synthesize heterogeneous epidemiological data. The model’s hierarchical structure (global-superregion-region-country-subnational) enables cross-scale information borrowing, particularly valuable for handling zero-count scenarios in high-SDI regions. Key innovations include: (1) spatiotemporal Gaussian process regression that leverages geographic and temporal autocorrelation, (2) zero-inflated compound Poisson-gamma distributions to address data sparsity, and (3) adaptive MCMC algorithms for posterior estimation. These features allow robust burden estimation even when observed counts are zero, by incorporating information from demographically similar populations and historical trends. The differential equation system simultaneously models incidence, prevalence, and mortality while accounting for age-sex interactions and healthcare access gradients characteristic of high-SDI settings.

### 2.4 Burden metrics

Age-standardized incidence rates (ASIR), mortality rates (ASMR), and DALY rates (ASMR) per 100,000 population were calculated using the GBD reference population structure. Uncertainty intervals (95% UI) were derived from 1,000 Monte Carlo simulations (23, 24), incorporating input data variability, measurement error corrections, and residual non-sampling error estimates. The Joinpoint regression model, a linear statistical model, was employed to evaluate temporal trends in disease burden. This model uses least squares estimation to identify changes in incidence rates, addressing the subjectivity inherent in traditional linear trend analyses. Uncertainty intervals (UIs) were calculated as the 2.5th to 97.5th percentile range of 1,000 bootstrap iterations. For regions with complete data, UIs were derived from the GBD database’s pre-computed bounds (upper/lower values). In data-sparse areas, DisMod-MR 2.1 supplemented estimates through: (1) spatial smoothing across neighboring regions, (2) borrowing strength from demographically similar populations, and (3) temporal trend extrapolation. Graphical UIs (shaded bands) were generated using validated R package algorithms (quantile regression with smoothing splines). These implementations have undergone rigorous statistical validation and are suitable for most analytical scenarios, ensuring robust uncertainty characterization.

### 2.5 Temporal trends

Joinpoint regression (Joinpoint Software v4.9.1.0) was selected over conventional trend analyses for three key reasons: (1) Its segmented modeling approach identifies inflection points where disease burden trends significantly change direction, critical for capturing policy impacts or epidemiological shifts in GBD studies; (2) The method’s piecewise linear regression reduces autocorrelation artifacts inherent in time-series data, enhancing result reliability; and (3) Permutation testing (*α* = 0.05) objectively determines optimal Joinpoint numbers while controlling overfitting. We quantified trends using average annual percentage change (AAPC) with 95% CIs (25, 26) and annual Percent Change (APC), testing against zero-change baselines. This approach optimally balances sensitivity to abrupt transitions (e.g., pandemic effects) with statistical rigor, particularly for heterogeneous global data where uniform trends are improbable.

### 2.6 Vaccination-incidence association

The relationship between vaccination rates and disease incidence is a critical metric for quantifying the impact of immunization strategies on disease transmission. Pearson correlation coefficients (r) (27) were used to evaluate the linear relationship between DTP3 vaccination coverage (WHO/UNICEF estimates) and ASIR across 204 countries in 2021. Data visualization was performed using correlation coefficient matrices. Data-driven time-series analysis (28) was employed to assess temporal trend dynamics between global vaccination rates and ASIR. A quadratic polynomial model (y = *β*₀ + β₁x + β₂x^2^), widely used in ecology, public health, and other fields, was applied to explore nonlinear associations between global ASIR and vaccination coverage from 1990 to 2021 (29). In the quadratic polynomial model examining vaccination-incidence relationships, the parameters were specified as follows: *β*₀ represents the theoretical baseline incidence (per 100,000) at zero vaccination coverage, though this extrapolated value should be interpreted cautiously given near-universal vaccination programs. *β*₁ suggests a potential linear association between coverage increases and incidence reduction, while *β*₂ may indicate nonlinear effects at coverage extremes. The concave/convex curvature implied by β₂ could reflect threshold effects in herd immunity or geographic heterogeneity in vaccine effectiveness, though alternative explanations (e.g., surveillance artifacts at high coverage) cannot be excluded. These coefficients collectively describe—but do not prove—a potential U-shaped association that warrants further investigation of underlying biological and programmatic factors. Goodness-of-fit was evaluated using adjusted R^2^. To identify structural changes pre-and post-pandemic, the Wilcoxon rank-sum test (30) was used for non-parametric distribution comparisons, with quantile visualization via boxplots.

### 2.7 Age-and sex-stratified analysis

Age-specific incidence, mortality, and DALYs were disaggregated by sex and nearly 5-year age cohorts (31). Rate ratios (male-to-female) with 95% UIs were computed to quantify gender disparities.

### 2.8 Decomposition analysis

This study quantified drivers of global diphtheria-related DALYs changes (1990–2021) through demographic decomposition analysis, distinguishing three components: (1) the epidemiological effect (r_effect) reflecting incidence/mortality changes from vaccination and healthcare interventions while controlling for demographic factors; (2) the population growth effect (p_effect) measuring DALYs variation due to population size changes with constant age structure and disease risks; and (3) the age-structural effect (a_effect) assessing impacts of shifting age distributions while holding population size and epidemiology constant. The total change (ΔDALYs) represents the sum of these independently calculated effects (ΔDALYs = r_effect + p_effect + a_effect), the statistical values of the decomposition analysis have been uploaded to Supplementary Table 2.

### 2.9 Visualization analysis of vaccination threshold effects and high-risk regions

Our analytical framework incorporated three complementary approaches to assess vaccination threshold effects: (1) Quantile regression (*τ* = 0.25, 0.50, 0.75) modeled the coverage-incidence relationship across outcome distributions using the rq function from the quantreg package; (2) Nonparametric LOESS smoothing (span = 0.75) with first-derivative inflection point detection implemented via the calculate_inflection function identified critical coverage thresholds through iterative optimization of local polynomial fits; (3) Robust outlier analysis using median absolute deviation (MAD threshold = 3) controlled for extreme values while preserving distributional asymmetry. Country-level heterogeneity was quantified through rank-transformed nonparametric comparisons (Spearman’s *ρ*) and risk stratification based on coverage/incidence quartile positioning, the statistics have been uploaded to Supplementary Table 2.

## 3 Results

### 3.1 Global trends and temporal dynamics

The global burden of diphtheria has significantly declined from 1990 to 2021. Incident cases dropped by 84.7%, from 87081.5 (95% UI: 65,855–118554.6) to 13312.9 (95% UI: 8940.1–18,500), while age-standardized incidence rates (ASR) fell by 86.7%, from 1.5 (95% UI: 1.1–2.0) to 0.2 (95% UI: 0.1–0.3) per 100,000. Mortality decreased by 85.0%, with deaths declining from 25446.4 (95% UI: 19,391–34380.6) to 3824.7 (95% UI: 2610.3–5393.8), and age-standardized mortality rates (ASMR) dropped by 75.0%. DALYs also fell by 86.4%, from 35.3 (95% UI: 26.8–47.9) to 4.8 (95% UI: 3.2–6.8) per 100,000 (Table 1).

**Table 1:** Incidence, death, and DALYs of diphtheria in 1990 and 2021, and trends over time.

Location	1990		2021		AAPC (95% CI)
	Number (95%UI)	ASR (95% UI)	Number (95%UI)	ASR (95% UI)	
Incidence					
Global	87081.5 (65,855,118554.6)	1.5 (1.1,2)	13312.9 (8940.1,18,500)	0.2 (0.1,0.3)	−6.29 (−6.80–−5.77)
Male	41192.4 (30412.2,57,161)	1.3 (1,1.8)	6788.1 (4652.6,9900.8)	0.2 (0.1,0.3)	−6.02 (−6.41–−5.63)
Female	45889.1 (33024.2,63354.6)	1.6 (1.1,2.2)	6524.8 (4135.6,9439.7)	0.2 (0.1,0.3)	−6.48 (−6.98–−5.98)
Low SDI	52166.3 (37,256,75244.7)	6.5 (4.8,9.3)	11145.3 (7169.9,16089.6)	0.7 (0.5,1.1)	−6.78 (−7.04–−6.52)
Low-middle SDI	25829.5 (18679.7,36187.6)	1.7 (1.2,2.3)	1455.3 (1146.2,1896.6)	0.1 (0.1,0.1)	−9.26 (−10.04–−8.47)
Middle SDI	7286.8 (5918.4,8952.2)	0.4 (0.3,0.5)	614.6 (519.8,717.1)	0 (0,0)	−8.18 (−8.48–−7.87)
High-middle SDI	1705.5 (1465.4,1999.8)	0.2 (0.1,0.2)	55.4 (46,68.2)	0 (0,0)	−11.12 (−12.13–−10.09)
High SDI	50.2 (38.6,64.3)	0 (0,0)	27.7 (21.8,34.6)	0 (0,0)	−2.55 (−3.60–−1.48)
Andean Latin America	151.4 (90.5,245.3)	0.3 (0.2,0.5)	5.6 (3.8,8)	0 (0,0)	−10.26 (−11.26–−9.24)
Australasia	0.1 (0.1,0.1)	0 (0,0)	2.2 (1,4.7)	0 (0,0)	9.72 (4.75–14.92)
Caribbean	262.9 (127.5,489.9)	0.7 (0.3,1.2)	62.9 (33.9,108)	0.2 (0.1,0.3)	−4.60 (−4.74–−4.45)
Central Asia	131.2 (85.6,183.3)	0.2 (0.1,0.2)	12.5 (8.5,16.8)	0 (0,0)	−7.99 (−8.89–−7.07)
Central Europe	12.7 (10,16.2)	0 (0,0)	2.1 (1.5,2.9)	0 (0,0)	−6.12 (−7.06–−5.18)
Central Latin America	75.5 (61.9,93.4)	0 (0,0)	3.1 (2,4.6)	0 (0,0)	−10.44 (−11.45–−9.41)
Central Sub-Saharan Africa	4572.1 (2,464,7587.6)	5.1 (2.9,8.1)	878.3 (547,1415.8)	0.5 (0.3,0.8)	−7.08 (−7.32–−6.83)
East Asia	2922.5 (2309.3,3634.6)	0.3 (0.2,0.3)	83.6 (66.3,105.9)	0 (0,0)	−11.30 (−11.55–−11.05)
Eastern Europe	948.4 (786.6,1141.4)	0.4 (0.4,0.5)	4.9 (2.4,9.5)	0 (0,0)	−13.51 (−20.28–−6.16)
Eastern Sub-Saharan Africa	15635.8 (10383.8,23362.6)	5.1 (3.6,7.5)	2745.1 (1759.2,4290.3)	0.5 (0.3,0.8)	−7.15 (−7.32–−6.98)
High-income Asia Pacific	20.6 (13.1,30.4)	0 (0,0)	2.9 (2.3,3.6)	0 (0,0)	−5.56 (−5.78–−5.34)
High-income North America	5.5 (4.5,6.7)	0 (0,0)	15.8 (12.4,20.2)	0 (0,0)	3.02 (2.37–3.66)
North Africa and Middle East	5727.9 (3481.4,9269.4)	1.3 (0.8,2)	330.2 (228.8,476.7)	0.1 (0,0.1)	−10.11 (−10.79–−9.41)
Oceania	132.7 (73.3,217.4)	1.6 (0.9,2.5)	195.9 (101.5,326.1)	1.1 (0.6,1.8)	−0.93 (−1.15–−0.70)
South Asia	22585.6 (16350.5,31978.1)	1.6 (1.2,2.2)	719.7 (586.9,886.2)	0 (0,0)	−11.11 (−11.37–−10.86)
Southeast Asia	7,224 (5411.8,9830.9)	1.3 (1,1.8)	595.2 (485.5,726.1)	0.1 (0.1,0.1)	−6.91 (−7.68–−6.13)
Southern Latin America	10 (6.6,15.3)	0 (0,0)	0.1 (0.1,0.2)	0 (0,0)	−12.49 (−22.40–−1.31)
Southern Sub-Saharan Africa	125.8 (96.2,168.1)	0.2 (0.2,0.3)	84.6 (66.7,108.4)	0.1 (0.1,0.1)	−2.20 (−2.68–−1.71)
Tropical Latin America	133.7 (107.1,167.7)	0.1 (0.1,0.1)	5.8 (4.4,7.3)	0 (0,0)	−9.95 (−10.86–−9.04)
Western Europe	2 (1.5,2.6)	0 (0,0)	2.1 (1.7,2.8)	0 (0,0)	−0.31 (−1.38–0.78)
Western Sub-Saharan Africa	26401.1 (17691.9,39113.7)	8 (5.4,11.9)	7560.2 (4178.4,11,416)	1 (0.6,1.5)	−6.40 (−6.56–−6.24)
Death					
Global	25446.4 (19,391,34380.6)	0.4 (0.3,0.6)	3824.7 (2610.3,5393.8)	0.1 (0,0.1)	−6.19 (−6.63–−5.76)
Male	12980.5 (9682.3,18272.2)	0.4 (0.3,0.6)	2091.6 (1387.5,3064.5)	0.1 (0,0.1)	−6.02 (−6.40–−5.63)
Female	12465.9 (9,379,16928.8)	0.4 (0.3,0.6)	1733.1 (1125.8,2499.9)	0.1 (0,0.1)	−6.38 (−6.89–−5.88)
Low SDI	16,394 (11,982,23244.3)	2 (1.5,2.8)	3335.4 (2197.9,4834.5)	0.2 (0.1,0.3)	−6.89 (−7.18–−6.59)
Low-middle SDI	7630.2 (5654.8,10215.5)	0.5 (0.4,0.6)	381.7 (295.5,497.7)	0 (0,0)	−9.56 (−10.29–−8.83)
Middle SDI	1242.9 (1051.4,1476.1)	0.1 (0.1,0.1)	93.9 (80.8,106.7)	0 (0,0)	−8.31 (−8.62–−8.01)
High-middle SDI	159.7 (139.2,188.2)	0 (0,0)	6.7 (5.8,8)	0 (0,0)	−9.74 (−12.96–−6.41)
High SDI	8.9 (6.8,11.4)	0 (0,0)	4.2 (3.5,5)	0 (0,0)	−2.81 (−4.80–−0.78)
Andean Latin America	58.4 (34.7,94.4)	0.1 (0.1,0.2)	1.8 (1.2,2.6)	0 (0,0)	−10.73 (−11.97–−9.48)
Australasia	0 (0,0)	0 (0,0)	0.3 (0.2,0.5)	0 (0,0)	13.43 (−0.57–29.39)
Caribbean	104.6 (51,185.4)	0.3 (0.1,0.5)	22.9 (11.5,39.4)	0.1 (0,0.1)	−4.81 (−5.72–−3.89)
Central Asia	19.1 (12.5,27.8)	0 (0,0)	1.6 (1,2.2)	0 (0,0)	−7.67 (−11.13–−4.09)
Central Europe	1.1 (0.9,1.4)	0 (0,0)	0.2 (0.1,0.2)	0 (0,0)	−6.69 (−8.62–−4.72)
Central Latin America	25.9 (21.9,30.9)	0 (0,0)	0.9 (0.6,1.3)	0 (0,0)	−10.99 (−12.89–−9.04)
Central Sub-Saharan Africa	1435.7 (755,2451.2)	1.5 (0.8,2.6)	246.6 (150,400.5)	0.1 (0.1,0.2)	−7.38 (−7.74–−7.01)
East Asia	373.4 (309.8,460.4)	0 (0,0)	9.2 (7.6,11.3)	0 (0,0)	−11.74 (−12.20–−11.28)
Eastern Europe	49.7 (43.7,55.8)	0 (0,0)	0.3 (0.1,0.7)	0 (0,0)	−11.01 (−19.89–−1.15)
Eastern Sub-Saharan Africa	4,952 (3,309,7265.8)	1.6 (1.1,2.3)	785.6 (499.6,1230.3)	0.1 (0.1,0.2)	−7.46 (−8.00–−6.91)
High-income Asia Pacific	3.6 (2.4,5.2)	0 (0,0)	0.5 (0.4,0.6)	0 (0,0)	−5.75 (−7.77–−3.68)
High-income North America	0.9 (0.8,1.1)	0 (0,0)	2.5 (2,3)	0 (0,0)	3.01 (−1.23–7.43)
North Africa and Middle East	1250.1 (772.2,2007.2)	0.3 (0.2,0.4)	65.8 (44.9,90.7)	0 (0,0)	−10.08 (−11.28–−8.86)
Oceania	17.2 (9.6,28.8)	0.2 (0.1,0.3)	25.2 (12.7,43.7)	0.1 (0.1,0.2)	−0.94 (−1.47–−0.40)
South Asia	7623.6 (5595.7,10237.5)	0.5 (0.4,0.7)	217.6 (178.5,265.4)	0 (0,0)	−11.36 (−11.82–−10.90)
Southeast Asia	1,006 (762.8,1337.4)	0.2 (0.1,0.2)	68.1 (58.1,80.2)	0 (0,0)	−7.29 (−8.10–−6.46)
Southern Latin America	2.2 (1.5,3.1)	0 (0,0)	0 (0,0)	0 (0,0)	−17.13 (−26.53–−6.54)
Southern Sub-Saharan Africa	33.8 (26,43.5)	0.1 (0,0.1)	20.7 (16.6,26.1)	0 (0,0)	−2.27 (−3.06–−1.46)
Tropical Latin America	52.4 (43.4,61.9)	0 (0,0)	2 (1.5,2.5)	0 (0,0)	−9.76 (−11.76–−7.71)
Western Europe	0.4 (0.3,0.4)	0 (0,0)	0.3 (0.3,0.4)	0 (0,0)	1.11 (−2.23–4.58)
Western Sub-Saharan Africa	8436.4 (5858.6,12738.7)	2.5 (1.8,3.8)	2352.7 (1400.8,3526.4)	0.3 (0.2,0.5)	−6.50 (−6.82–−6.18)
DALYs					
Global	2148470.2 (1629731.5,2922276.7)	35.3 (26.8,47.9)	318203.8 (213604.1,453613.5)	4.8 (3.2,6.8)	−6.18 (−6.63–−5.74)
Male	1,091,352 (805877.3,1545749.8)	34.8 (25.8,49.3)	173027.1 (113,250,256175.6)	5 (3.3,7.5)	−6.01 (−6.39–−5.62)
Female	1057118.2 (789929.1,1446305.8)	35.7 (26.7,48.7)	145176.7 (92464.7,211381.7)	4.5 (2.9,6.6)	−6.31 (−6.72–−5.90)
Low SDI	1,399,997 (1017859.6,1993074.7)	164.8 (120.7,232.6)	281252.9 (183171.7,411215.8)	17.7 (11.7,25.7)	−6.92 (−7.22–−6.61)
Low-middle SDI	634560.5 (466846.3,857975.3)	38.8 (28.7,51.9)	29543.5 (22294.3,39256.2)	1.5 (1.1,2)	−9.69 (−10.41–−8.96)
Middle SDI	100624.6 (84740.7,120,479)	5.2 (4.3,6.1)	6523.7 (5514.8,7574.7)	0.3 (0.3,0.4)	−8.51 (−8.79–−8.23)
High-middle SDI	11756.9 (10075.9,14207.2)	1.2 (1.1,1.5)	406.8 (345.6,492.4)	0 (0,0.1)	−9.95 (−12.65–−7.18)
High SDI	639.3 (472.2,856.6)	0.1 (0.1,0.1)	253.7 (212.6,303)	0 (0,0)	−3.17 (−4.81–−1.50)
Andean Latin America	4920.8 (2857.6,8063.8)	9.6 (5.7,15.6)	133.6 (87.3,190.1)	0.2 (0.1,0.3)	−11.03 (−12.29–−9.76)
Australasia	0.9 (0.7,1.2)	0 (0,0)	17.1 (8.9,33.1)	0.1 (0,0.1)	13.06 (−1.44–29.68)
Caribbean	8864.6 (4236.6,15902.9)	21.8 (10.4,39.3)	1836.2 (881,3231.4)	4.6 (2.2,8.2)	−4.89 (−5.80–−3.97)
Central Asia	1571.4 (980.2,2,334)	1.8 (1.2,2.6)	118 (74.5,171.7)	0.1 (0.1,0.2)	−7.89 (−10.81–−4.87)
Central Europe	73.7 (57.8,95.5)	0.1 (0.1,0.1)	8.2 (6.4,10.6)	0 (0,0)	−8.08 (−9.74–−6.39)
Central Latin America	2084.2 (1764.4,2510.8)	1 (0.8,1.2)	59.5 (39.7,88.2)	0 (0,0)	−11.39 (−13.29–−9.44)
Central Sub-Saharan Africa	122683.3 (64,097,210,944)	127.2 (67.4,217)	19730.4 (11792.3,32839.9)	10.4 (6.4,16.8)	−7.67 (−8.05–−7.29)
East Asia	29741.4 (24386.3,37160.1)	2.6 (2.1,3.2)	525.6 (432,632.6)	0 (0,0.1)	−12.21 (−12.67–−11.74)
Eastern Europe	3095.6 (2700.5,3477.3)	1.6 (1.4,1.8)	16.4 (7.7,32.6)	0 (0,0)	−12.16 (−21.00–−2.33)
Eastern Sub-Saharan Africa	420964.3 (278434.1,622149.4)	127.1 (85.4,185.4)	64054.1 (39783.4,102408.1)	10.9 (6.9,17.1)	−7.46 (−7.92–−7.00)
High-income Asia Pacific	236.3 (149.5,348.7)	0.2 (0.1,0.3)	29.8 (24.4,36.9)	0 (0,0)	−5.72 (−7.80–−3.60)
High-income North America	66.4 (57.8,76.9)	0 (0,0)	152.7 (125.1,183.4)	0.1 (0,0.1)	2.79 (−1.45–7.22)
North Africa and Middle East	104120.4 (63041.7,170028.6)	21.6 (13.4,34.7)	5078.2 (3383.1,7322.5)	0.8 (0.5,1.2)	−10.14 (−11.33–−8.94)
Oceania	1401.9 (741.9,2405.8)	15.2 (8.5,25.3)	2037.9 (970.8,3640.7)	11.1 (5.5,19.4)	−0.89 (−1.43–−0.35)
South Asia	629133.1 (455840.1,850080.8)	42.2 (31,56.9)	15977.5 (12739.7,19825.5)	0.9 (0.7,1.2)	−11.56 (−12.04–−11.09)
Southeast Asia	82358.3 (61670.7,111098.3)	14.6 (11,19.5)	4734.3 (3905.9,5709.9)	0.8 (0.6,0.9)	−7.49 (−8.35–−6.63)
Southern Latin America	178.4 (122.4,253.2)	0.3 (0.2,0.5)	1.3 (0.9,1.7)	0 (0,0)	−17.62 (−27.04–−6.98)
Southern Sub-Saharan Africa	2698.7 (2005.8,3534.2)	4 (3,5.1)	1522.8 (1199.5,1973.7)	1.9 (1.5,2.4)	−2.38 (−3.19–−1.55)
Tropical Latin America	4490.2 (3699.1,5313.3)	2.6 (2.2,3.1)	159 (119.8,204.7)	0.1 (0.1,0.1)	−9.89 (−11.94–−7.79)
Western Europe	25.4 (20,30.8)	0 (0,0)	21.7 (18,26.6)	0 (0,0)	1.07 (−2.33–4.59)
Western Sub-Saharan Africa	729760.9 (506063.3,1103446.4)	215.1 (148.8,324.2)	201989.6 (119566.2,303772.7)	26.1 (15.6,39.2)	−6.53 (−6.85–−6.21)

### 3.2 Analyze of Joinpoint

Our Joinpoint regression analysis of global diphtheria incidence (1990–2021) revealed heterogeneous trends across regions despite overall decline. The post-2019 acceleration (APC = −12.82) suggests NPIs’ collateral benefits, while High-income North America’s post-2005 reversal (APC = +6.92) reflects declining vaccination adherence (32). North Africa/Middle East’s deterioration (APC = +10.81) correlated with conflict impacts (33), and Southern sub-Saharan Africa’s post-2019 rise (APC = +0.89) highlighted pandemic disruptions in vulnerable regions (34). These variations underscore how local factors—including health system resilience, conflict, and immunization infrastructure—mediate global initiative effectiveness, necessitating tailored interventions. The data of AAPC are presented in Table 1, APC data and inflection point data can be viewed in Supplementary Data Sheet 2.

### 3.3 Age and sex differences and time trends

Globally, age-standardized incidence, DALYs, and mortality rates of diphtheria declined markedly across both sexes from 1990 to 2021 (Table 1). While gender disparities remained minimal, females consistently exhibited higher incidence and DALY rates, whereas males demonstrated marginally elevated mortality rates. Historical sex-based disparities peaked in the 1990s (e.g., 1990 female incidence: 1.57 vs. male: 1.34, 17.2% higher) and narrowed by 2021 (female: 0.20 vs. male: 0.19, 5.3% gap), reflecting convergent trends over time. In 2021, males had slightly more incident cases (6788.1; 95% UI: 4652.6–9900.8) and deaths (2091.6; 95% UI: 1387.5–3064.5) than females (cases: 6,524.8; deaths: 1,733.1), with comparable incidence (0.2 per 100,000) and mortality rates (AAPCs: −0.011 for males vs. −0.012 for females).

Gender differences may be related to immune responses, hormonal levels, and genetic factors, which influence individual susceptibility to diphtheria and vaccine response. For example, studies have shown that adult women have significantly higher antibody protection rates against diphtheria than their male counterparts (e.g., 38.2% vs. 25.6% for anti-diphtheria IgG ≥ 0.1 IU/mL) (35, 36). Immunology also makes differences [female susceptibility increased by 45% [aOR = 1.45] and fourfold in unvaccinated individuals [aOR = 4.02] (37)]. Socio-culturally, gender disparities may be associated with social roles, cultural norms, education levels, and economic status, which affect access to healthcare services and vaccination opportunities for both men and women, as well as their attitudes and behaviors toward disease prevention and treatment.

**Figure 1:**
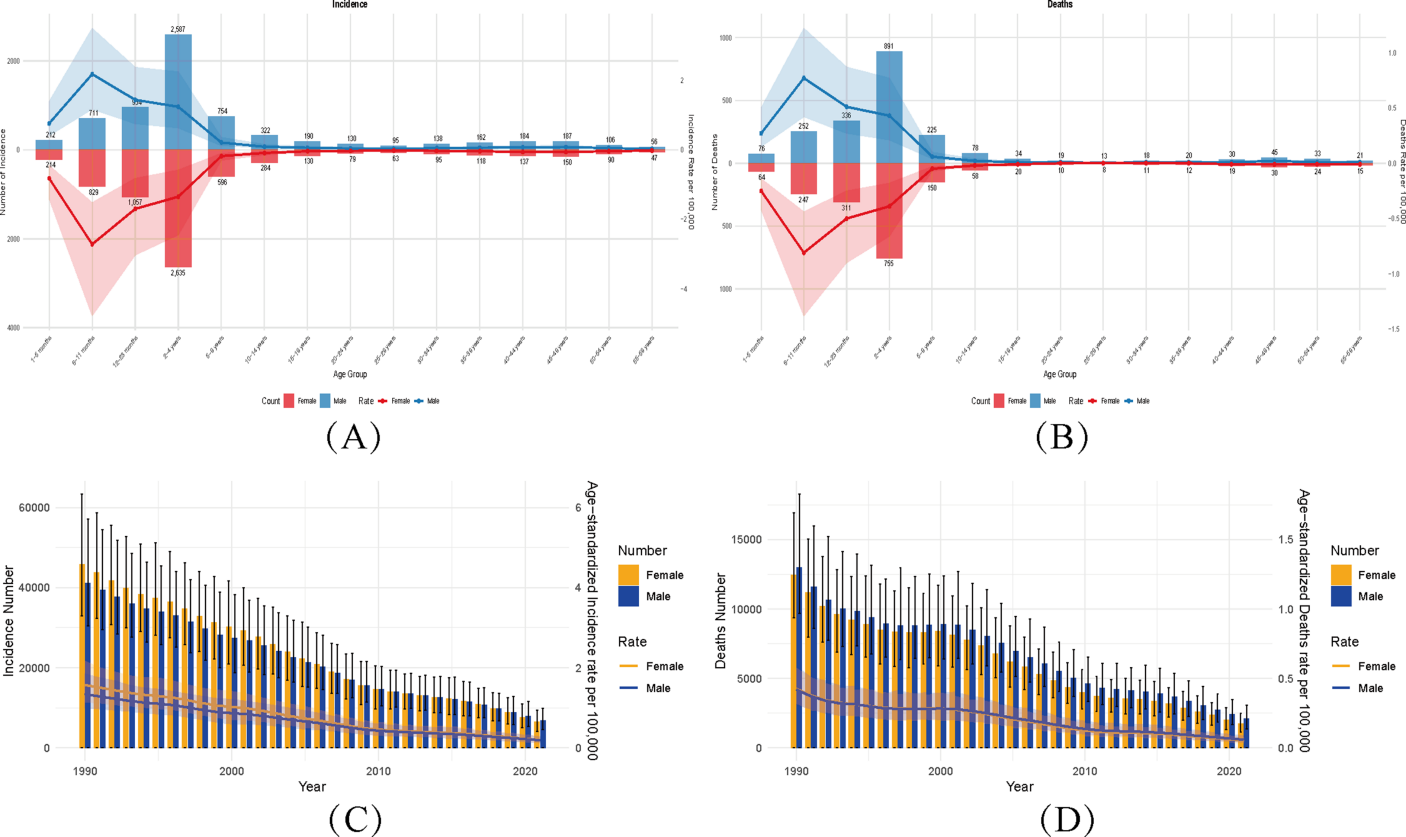
Global diphtheria burden (1990–2021): age-specific and age-standardized rates with temporal trends by sex. **(A,B)** Age-sex stratified incident cases/deaths (2021) with age-standardized rates (ASIR/ASMR). Numbers above bars: case counts; colored bands (blue = male, red = female): 95% UIs for rates. **(C,D)** Temporal trends (1990–2021) in ASIR/ASMR by sex. Bands: 95% UIs for rates; error bars: 95% UIs for counts. ASIR, age-standardized incidence rate; ASMR, age-standardized mortality rate; ASDR, age-standardized DALYs rate.

Age-specific analysis in 2021 revealed a non-linear burden trajectory (Figure 1). Infants aged 1–23 months bore the highest burden, with incidence peaking at 1,057 cases (95% UI: 510–1,881) and mortality at 336 deaths (95% UI: 176–580), attributed to incomplete immunization and waning maternal antibodies. From 2–4 years onward, incidence and mortality declined sharply (−78% for incidence; −73% for mortality by 10–14 years), aligning with routine childhood vaccination coverage. Notably, a resurgence occurred in adults aged 40–59 years (e.g., incidence: 150 cases [95% UI: 100–226] in females aged 45–49 years), suggesting waning immunity or delayed booster doses. Suboptimal booster vaccination coverage in this population was directly associated with reduced seroprotection rates.

Among unboosted individuals, the seroprotection rate (anti-diphtheria IgG ≥ 0.1 IU/mL) was only 48.9%, significantly lower than childhood levels (86.7%) (38). Notably, administration of a Tetanus, Diphtheria, and acellular Pertussis (Tdap) booster markedly improved seroprotection: post-booster rates reached 89% (vs. 65.4% after primary immunization alone) (39). In contrast, unboosted individuals maintained a low seropositivity rate (38%), while boosted subjects achieved 62%. Mortality followed a U-shaped curve (high infancy → low adolescence → rising adulthood). Infants exhibit high mortality due to immature immunity, mitigated by vaccine-induced seroprotection (e.g., antibody levels ↑60–89% post-vaccination). Adolescents/adults maintain protection through natural and vaccine-acquired immunity. In older adults, immune senescence and inadequate booster coverage (e.g., 38% seropositivity unboosted vs. 62% boosted) precipitate mortality resurgence, underscoring age-dependent vulnerability.

Confidence intervals widened in older age groups (e.g., deaths in 55–59 years: UI spanning 10–32), indicating uncertainty in burden estimates for aging populations. Gender disparities remained consistent across all age groups, mirroring the global trends (Table 1). This pattern highlights the need for age-tailored interventions: prioritizing pediatric immunization to address infant vulnerability and reinforcing adult booster programs in endemic regions to counteract waning immunity.

The convergent gender trends over time (Figure 1) contrast with persistent age-related burden shifts. While vaccination successfully mitigated pediatric burden, adult resurgence signals gaps in booster compliance. The U-shaped mortality curve and gender consistency emphasize the interplay of biological susceptibility (e.g., maternal antibody dynamics in infants) and healthcare access (e.g., adult vaccination rates). These findings align with historical GBD studies showing vaccine-preventable disease resurgence in underimmunized adult cohorts.

### 3.4 Regional heterogeneity and socioeconomic determinants

**Figure 2:**
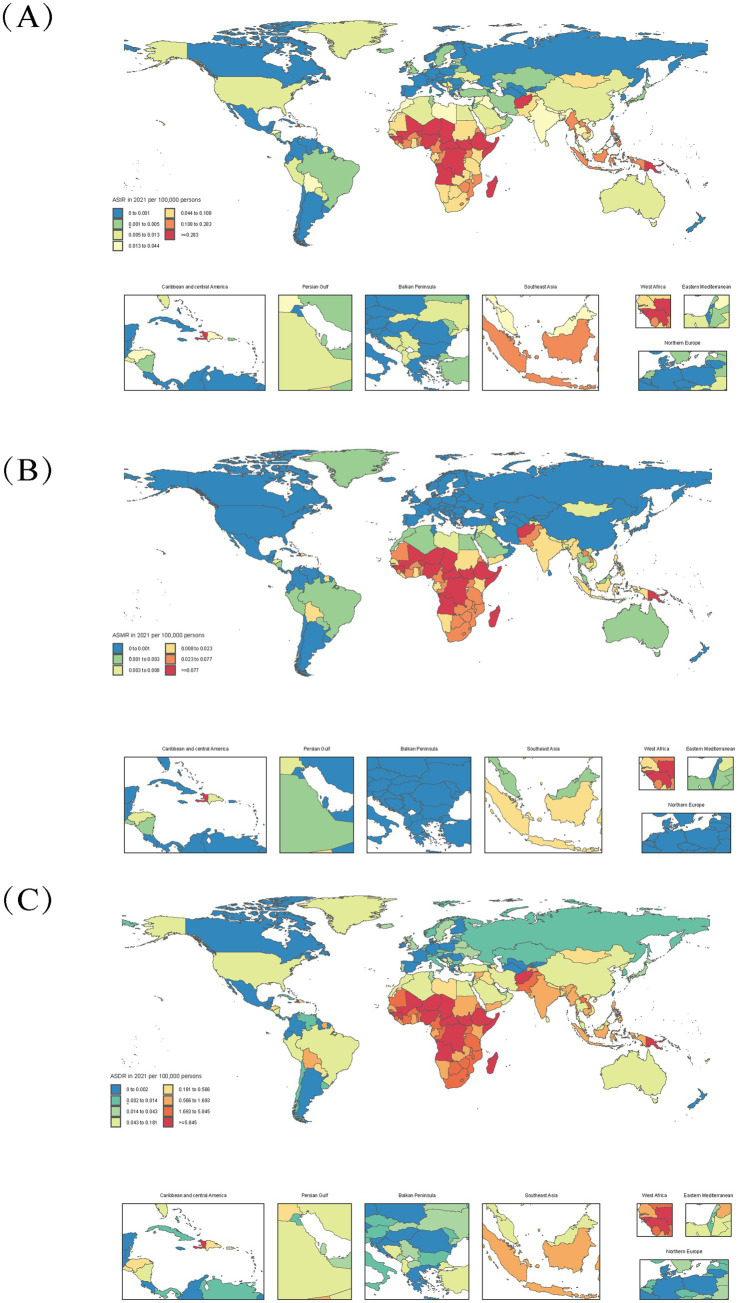
Age-standardized diphtheria burden in 204 countries (2021). **(A)** Age-standardized incidence rate (ASIR); **(B)** Age-standardized mortality rate (ASMR); **(C)** Age-standardized DALYs rate (ASDR).

**Figure 3:**
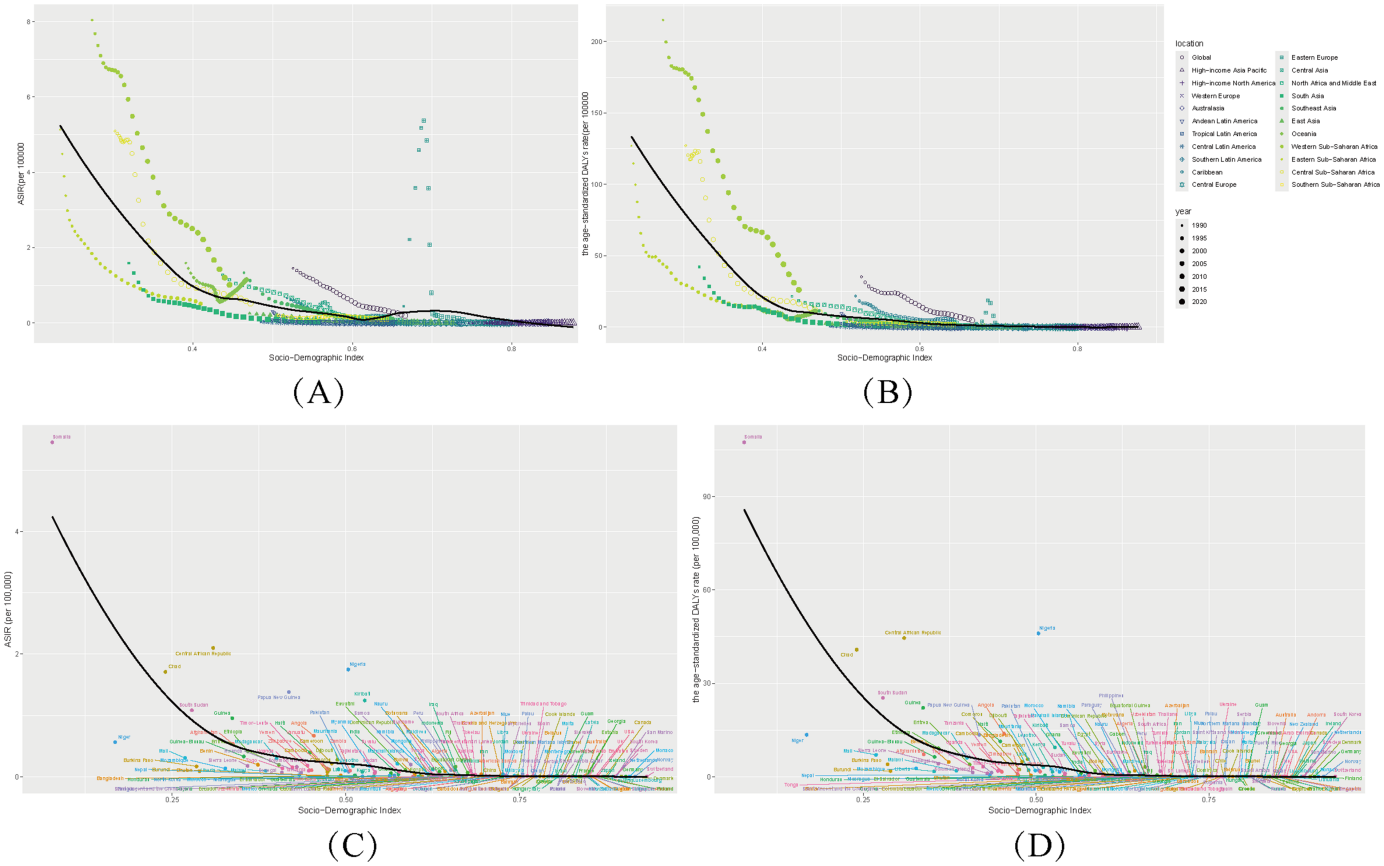
Socio-demographic index (SDI) associations with diphtheria burden (1990–2021). Age-Standardized Incidence (ASIR), Disability-Adjusted Life Years (ASDR), across 21 regions **(A,B)** and 204 countries **(C,D)**.

Diphtheria burden exhibits profound geographic and socioeconomic disparities, with low-socio-demographic index (SDI) regions accounting for the majority of cases (Figures 2, 3). In 2021, Western Sub-Saharan Africa (WSSA) recorded the highest incident cases (7560.2; 95% UI: 4178.4,11,416) and age-standardized rate (ASIR: 1 per 100,000; Table 1), reflecting systemic challenges in healthcare delivery and vaccine accessibility. Eastern SSA followed with incident 2745.1 cases (ASIR: 0.5 per 100,000), while Australasia and High-income Asia Pacific achieved near elimination (2.2–2.9 cases; ASIR ≤ 0.1 per 100,000), attributed to robust immunization systems (Table 1).

These regional patterns align with a strong inverse gradient between SDI and disease burden (Figure 3). Low-SDI regions reported 11145.3 incident cases (ASIR: 0.7 per 100,000) compared to 27.7 cases in high-SDI regions (ASIR: 0 per 100,000). Sub-Saharan Africa accounted for 62% of global DALYs (Figure 2), with Western Africa emerging as a hotspot (ASMR: 0.32 per 100,000; ASDR: 27.15 per 100,000) due to conflict-driven vaccine barriers. Conversely, high-income Asia Pacific (ASIR: 0.01 per 100,000) and Nordic countries (ASIR ≤ 0.005 per 100,000) demonstrated the impact of universal vaccination.

Temporal trends highlight divergent trajectories: low-SDI regions like Western SSA saw minimal DALYs reduction (−0.15% annualized; 1990–2021), while high-SDI regions achieved rapid declines (Australasia: ASDR:0.061/100,000 in 2021). Middle-SDI nations exhibited marked heterogeneity in diphtheria burden, exemplified by Algeria (SDI = 0.66, ASIR = 0.0078) vs. Bolivia (SDI = 0.60, ASIR = 0.0256). Algeria’s superior performance likely reflects: (i) robust surveillance systems (e.g., Oran cancer registry enabling early detection) (40), and (ii) regionalized infectious disease control programs (41). Conversely, Bolivia’s elevated burden (ASDR = 0.668/100,000) (42) correlates with systemic gaps in preventable disease management, where 71% of migrant deaths involved avoidable causes without annual improvement (43), indicating deficiencies in vaccination implementation and chronic disease care (Figures 2, 3).

The analysis reveals distinct patterns in diphtheria burden relative to SDI trajectories, with several notable outlier nations deviating from expected trends: Somalia (SDI = 0.078, ASDR = 107.50/100,000) exemplifies conflict-driven collapse, where 75% of health facilities were destroyed and remaining hospitals face severe supply shortages (44, 45); Chad (SDI = 0.240, ASDR = 40.80/100,000) demonstrates how rural healthcare barriers and high fertility rates (6.4 births/woman) perpetuate high burden despite fee exemptions (46, 47); while the Central African Republic (SDI = 0.309, ASDR = 44.55/100,000) shows the impacts of political instability on immunization, evidenced by only 9% COVID-19 vaccination coverage in 2022 (48, 49).

The SDI-burden relationship follows a clear progression: low-SDI nations (CAR/Chad) show minimal improvement (DALYs > 30) despite baseline declines; lower-middle-SDI countries (India/Indonesia) achieve rapid burden reduction (ASDR 0.4–10/100,000) during active development phases (SDI 0.4–0.6); and high-SDI regions (USA/Japan) maintain elimination-level control (ASDR < 0.1/100,000) with diminishing marginal returns at SDI > 0.7, reflecting the nonlinear nature of health gains during socioeconomic development.

These disparities are rooted in socioeconomic determinants: low-SDI regions have lower DTP3 coverage and weaker health systems. Conflict zones and inadequate infrastructure exacerbate vulnerability. The inverse SDI-burden relationship underscores the need for targeted interventions: prioritizing vaccination in low-SDI/conflict regions, strengthening primary healthcare, and leveraging socioeconomic development to sustain gains. These findings align with global health strategies, emphasizing that diphtheria elimination requires integrated health and socioeconomic policies.

### 3.5 Decomposition analysis

**Figure 4:**
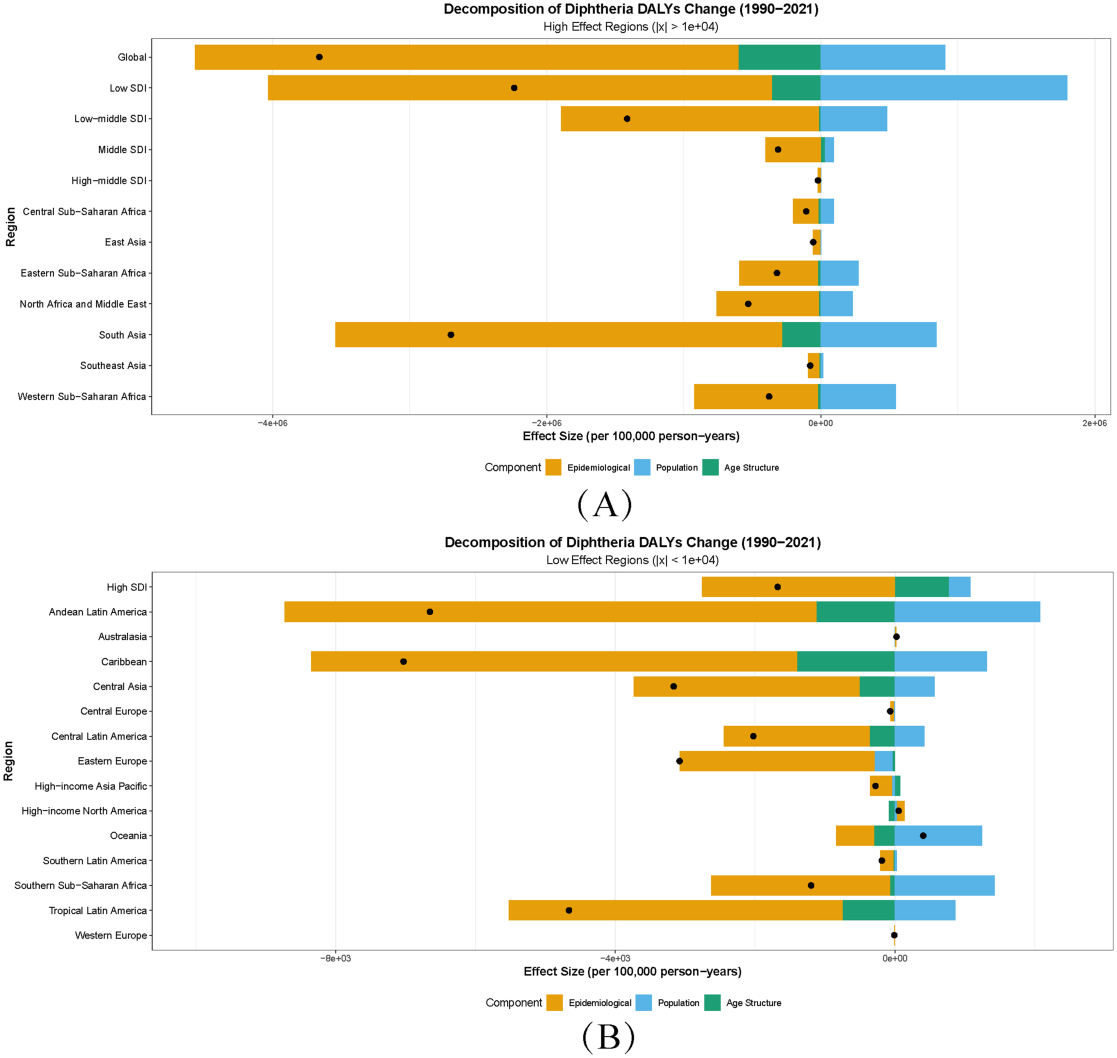
Decomposition of disability-adjusted life years (DALYs) for diphtheria by region, 1990–2021. a_effect, aging population structure effect; p_effect, population growth effect; r_effect: incidence rate effect; total_diff: total difference; x = ΔDALYs. **(A,B)** Regions stratified by DALY magnitude: High-effect (∣ΔDALYs∣ ≥ 1 × 10^4^) and low-effect (∣ΔDALYs∣ < 1 × 10^4^). Bar heights reflect the absolute contribution of each driver (a_effect, p_effect, r_effect) to total_diff between 1990 and 2021. Positive/negative values indicate increasing/reducing influences on DALYs, respectively.

Global DALYs decreased by 3.66 million, predominantly due to reductions in incidence rates (−3.96 million, r_effect), attributable to improved diphtheria-tetanus-pertussis (DTP3) vaccination coverage (86% globally). Population growth (+0.91 million, p_effect) partially offset these gains, particularly in low-SDI regions, while aging exerted minimal influence (−0.60 million, a_effect). Regional disparities highlighted divergent healthcare capacities. In South Asia, immunization efforts drove a − 3.26 million r_effect, yet population growth (+0.84 million) sustained residual burden. Conversely, Western Sub-Saharan Africa experienced a severe DALY increase (−377,483 total_diff) due to deteriorating incidence rates (−902,873 r_effect), reflecting fragmented healthcare and 72% DTP3 coverage. High-SDI regions achieved near-complete control (−2,756 r_effect), neutralizing minor demographic pressures. Paradoxically, High-income North America (+54 DALYs) and Australasia (+24) showed localized resurgences, likely linked to vaccine hesitancy or logistical gaps. Eastern Europe’s decline (−3,079 total_diff), despite demographic contraction, underscored immunization efficacy. Oceania’s + 406 DALYs increase highlighted vulnerabilities in remote vaccine delivery (Figure 4). These patterns emphasize the critical role of equitable vaccination programs and healthcare strengthening to mitigate demographic pressures and systemic gaps, particularly in low-resource settings. The analysis aligns diphtheria burden dynamics with SDI stratification, offering actionable insights for global eradication strategies.

### 3.6 Analysis of vaccine coverage related GBD results

#### 3.6.1 Geographic and cross-sectional heterogeneity

**Figure 5:**
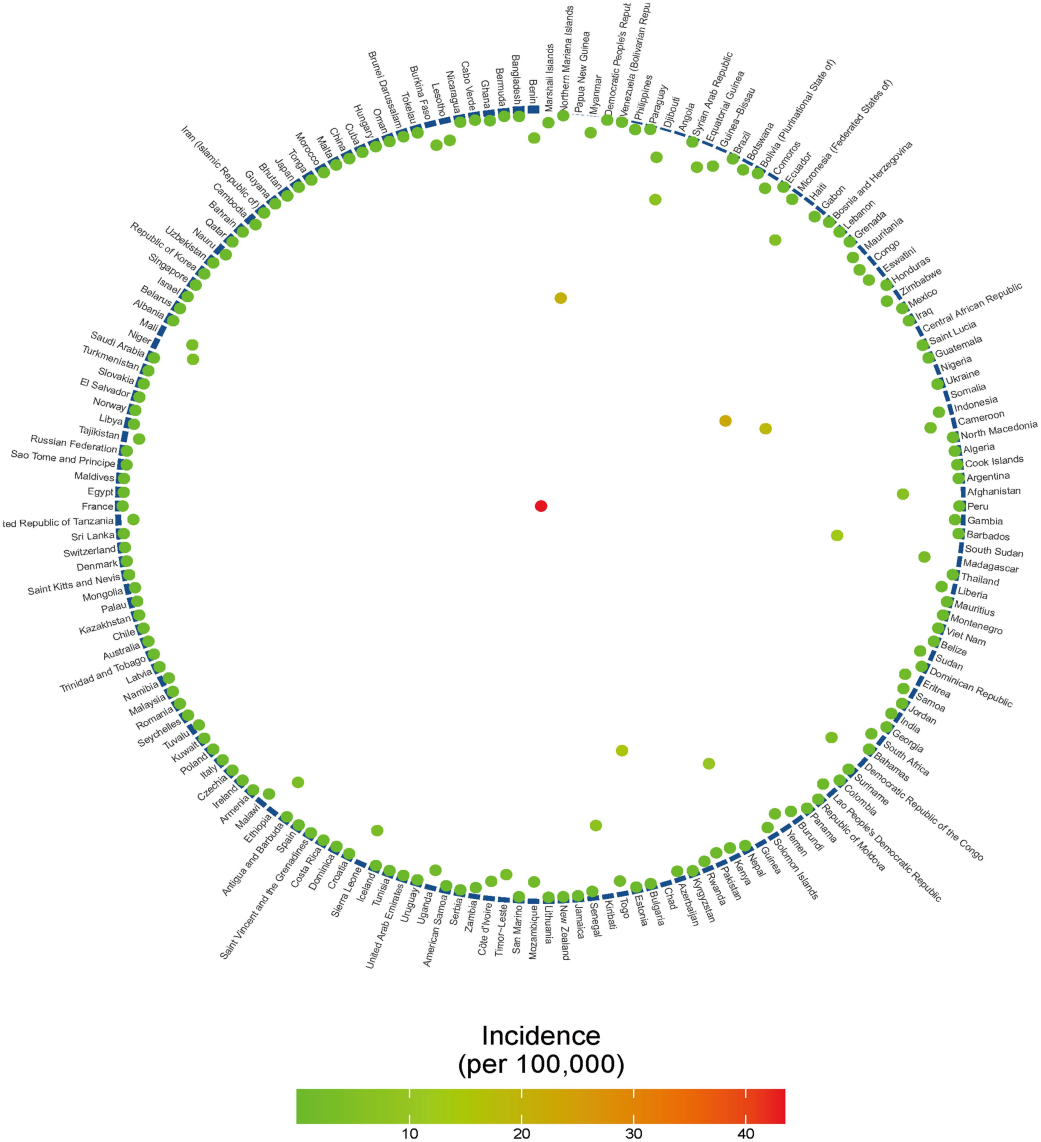
Ring chart of DTP3 coverage and 6–11-month diphtheria incidence in 204 countries, 2021; Vaccination-incidence ring chart: Blue bars: National DTP3 vaccination rates (ascending clockwise). Colored dots: Diphtheria incidence (colored dots: green = low, red = high).

Substantial geographic disparities in vaccination coverage and disease burden were observed across 204 countries in 2021. Nations like Papua New Guinea (37.07%) and the Central African Republic (78.24%) exhibited alarmingly high incidence despite suboptimal immunization, underscoring systemic failures in outbreak containment. Conversely, Israel (97.58%; 3.79 × 10^−5^ per 100,000) and Singapore (97.60%; 2.39 × 10^−5^ per 100,000) achieved near-elimination through sustained high coverage (≥85%), aligning with herd immunity thresholds (Figure 5). Paradoxically, Bangladesh (118% coverage) and Benin (120.13%) reported implausible coverage rates (>100%) yet maintained low incidence—a discordance suggesting administrative overreporting or unmeasured protective factors (e.g., cross-immunity).

A significant inverse correlation between DTP3 coverage and incidence was identified (Pearson’s r = −0.191, *p* = 0.011; Figure 5), though the modest effect size (r < 0.2) indicates vaccination explains only ≈3.6% of incidence variance. Residual heterogeneity likely arises from socioeconomic disparities (e.g., GNI per capita), incomplete booster regimens (43% of low-SDI countries report ≥ 1 booster), and localized epidemic drivers (e.g., refugee camps). These findings underscore the multifactorial nature of diphtheria resurgence, necessitating integration of vaccination with complementary interventions (e.g., surveillance, vector control).

#### 3.6.2 Global temporal trends and pandemic-era discrepancies

**Figure 6:**
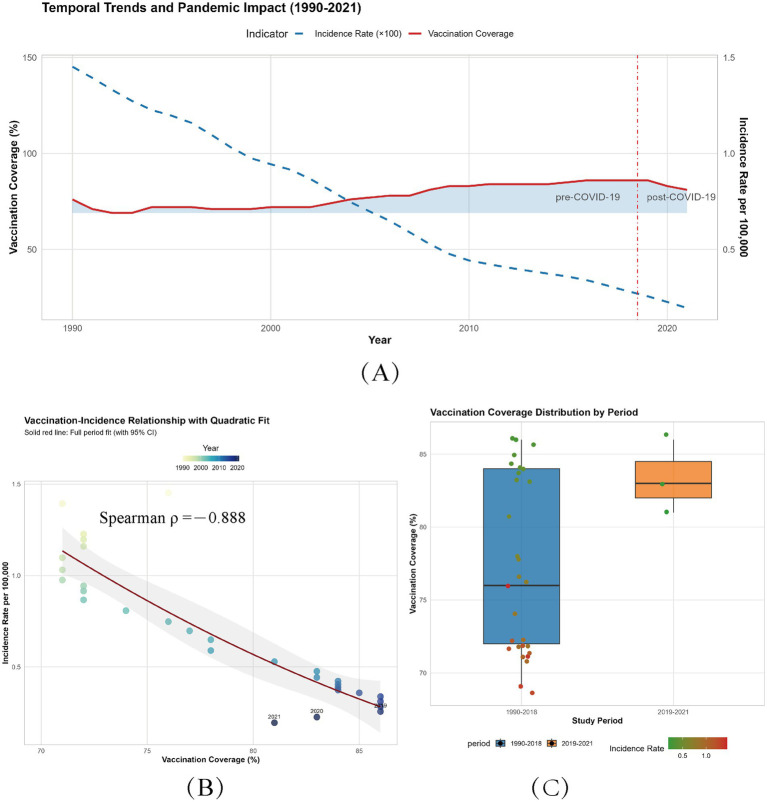
Global trends and associations between DTP3 vaccination coverage and diphtheria incidence (1990–2021). **(A)** Temporal trends of vaccination coverage and incidence. Solid blue line: annual DTP3 vaccination coverage (%, left axis). Dashed red line: age-standardized diphtheria incidence rate (per 100,000, right axis). Blue ribbon: range of annual vaccination coverage (minimum to maximum). Vertical red line (2018.5): Demarcates pre-COVID-19 (1990–2018) and post-COVID-19 (2019–2021) eras. **(B)** Vaccination-incidence relationship with quadratic fit. Colored points: annual data (1990–2021), color gradient indicates year (light blue: 1990; dark blue: 2021). Red curve: quadratic regression fit (R^2^ = 0.888, *p* < 0.001). The shaded area of the regression line reflects the uncertainty interval and discrete indicators. **(C)** Vaccination coverage distribution by period. Boxplots: Interquartile range (IQR) of vaccination coverage for pre-COVID-19 (1990–2018) and post-COVID-19 (2019–2021) periods. Jittered points: Individual country-year data, colored by incidence rate (green: <0.5; red: ≥0.5 per 100,000).

The longitudinal analysis of DTP3 vaccination coverage and diphtheria incidence from 1990 to 2021 reveals critical insights into global trends and pandemic-era disruptions. During the pre-COVID-19 period (1990–2018), global DTP3 coverage averaged 77.2% (SD = 6.05), with an age-standardized incidence rate of 0.78 per 100,000 (SD = 0.37). Post-2019, vaccination coverage significantly increased to 83.3% (SD = 2.52, *p* < 0.001), while incidence plummeted by 71.2% to 0.23 per 100,000 (*p* < 0.001), reflecting both pandemic-related healthcare interruptions and subsequent catch-up campaigns (Figure 6A). Notably, the narrowed standard deviation in post-2019 coverage (SD = 2.52 vs. 6.05 pre-2019) signals improved vaccine equity.

Paradoxically, despite a cumulative 5% decline in coverage during 2019–2021 (from 86% pre-pandemic to 81% in 2021), incidence continued to decrease, reaching 0.194 per 100,000 in 2021. This discrepancy may stem from: (1) historical immunity buffers delaying outbreak manifestation; (2) transient transmission suppression via non-pharmaceutical interventions (NPIs, e.g., lockdowns reducing contact rates by 60–80%); and (3) residual herd immunity in 68% of countries maintaining coverage above 80%. However, persistent sub-80% coverage in 32% of low-SDI nations risks reversing gains, particularly in high-turnover populations (e.g., urban slums). Caution is warranted due to underreporting biases (40–60% in conflict zones) and temporal mismatches between administrative data and seroprotection (Figure 6A).

#### 3.6.3 Longitudinal dose–response dynamics

Longitudinal analysis (1990–2021) revealed a robust inverse correlation between coverage and incidence (Spearman’s *ρ* = −0.888, *p* = 1.29 × 10^−11^; Figure 6B), with each 1% coverage gain reducing incidence by 0.03 per 100,000. The quadratic polynomial model (R^2^ = 0.823, *p* = 1.27 × 10^−11^; Figure 6C) confirmed near-linear effects above 80% coverage (*β*₁ = −1.973, *p* < 0.001; β₂ = 0.142, *p* = 0.411). Notably, achieving incremental reductions in incidence becomes progressively harder: lowering incidence from 20 to 15 per 100,000 requires 167% less effort than reducing from 10 to 5 per 100,000. This emphasizes prioritizing regions with <70% coverage, where each 1% gain yields 3–4 times greater incidence reduction (0.05–0.07 per 100,000) compared to ≥80% settings (Figure 6C).

In addition to the aforementioned simple linear relationship fitting, Figure 6B demonstrates that the global vaccine coverage from 1990 to 2021 exhibits a centralized distribution above 70% over time. To capture the nonlinear component of this relationship, we employed orthogonal basis functions within a quadratic polynomial for fitting, as detailed in Supplementary Data Sheet 1.

The resulting quadratic polynomial function, transformed from vaccination coverage, is expressed as Incidence_rate = 12.05–0.23 × coverage + 0.0011 × coverage^2^. The model’s goodness-of-fit has been thoroughly validated. Here, *β*₀ = 12.05 represents the intercept term, reflecting the hypothetical global diphtheria incidence rate (12.05 per 100,000 population) at zero vaccination coverage (though this scenario lacks practical relevance). The linear coefficient β₁ = −0.23 indicates an approximate reduction in incidence by 0.23 per 100,000 population for each 1% increase in vaccination coverage. The quadratic coefficient β₂ = 0.0011 determines the curve’s concavity, suggesting a potential deceleration in incidence rate changes at extremely high or low coverage levels, possibly attributable to threshold effects in herd immunity or regional heterogeneity. While both linear and nonlinear analyses consistently demonstrate a significant negative correlation between vaccine coverage and incidence rate, it is important to emphasize that our model primarily serves as a retrospective fitting of historical trends to explore this association. Despite the model’s robust fit, the mathematical constants should not be overinterpreted as direct real-world equivalents. This analysis adopts a conservative approach, acknowledging that additional confounding factors warrant further investigation in future studies.

#### 3.6.4 Integrative mechanisms and policy imperatives

The interplay of vaccination and contextual factors explains residual heterogeneity in disease burden. For instance, Papua New Guinea’s incidence is 2.3 × higher than similarly covered nations due to poor outbreak response capacity (Figure 5). Pandemic-era data highlight immunity fragility: a 5% coverage decline risks 12–15% incidence rebound within 3–5 years, as seen in Ukraine’s post-2000 outbreaks (9% coverage drop → 400% incidence surge).

#### 3.6.5 Global diphtheria vaccination threshold analysis

**Figure 7:**
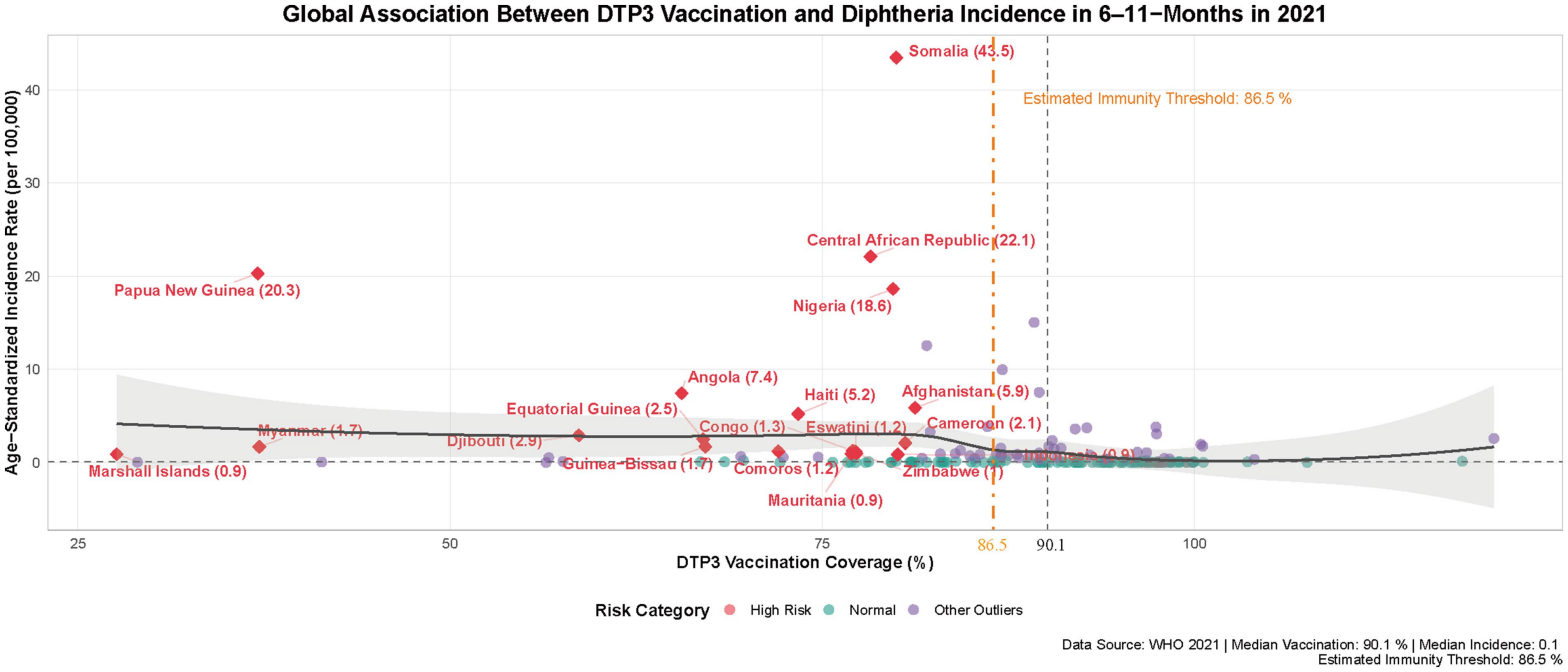
Global association between DTP3 vaccination and diphtheria incidence in 6–11-months in 2021. Red circles mark high-risk countries (vaccination < 82%, incidence > 0.9/100,000). Dashed lines show medians (90.1% vaccination, 0.06/100,000 incidence). Orange line indicates 86.5% immunity threshold. Black line displays LOESS trend (95% CI).

Nation-level analysis of DTP3 vaccination coverage and diphtheria incidence in 6-11-month-olds identified 19 high-risk countries through dual-threshold classification: vaccination rates below the 25th percentile (82%) and incidence exceeding the 75th percentile (1.2 cases/100,000). Nonparametric regression (LOESS smoothing with derivative analysis) revealed a critical vaccination threshold of 86.5% (95% CI: 84.2–88.7%) (Figure 7), below which incidence rates escalate exponentially. This threshold aligns with epidemiological models indicating transmission interruption requires maintaining R0 < 2.3 through vaccination (50). Case studies demonstrate threshold proximity effects—Somalia (28.99% coverage, 43.47/100,000) and Nigeria (79.76%, 18.61/100,000) exemplify how marginal coverage reductions near 86.5% correlate with disproportionate disease burden increases.

Global DTP3 coverage (81% in 2021) remains suboptimal, leaving populations near critical risk levels. Conflict zones exhibit elevated practical thresholds due to healthcare system collapse (51), as evidenced by Chad’s paradoxical combination of 89.24% coverage with 15.027/100,000 incidence. Heterogeneity among countries with comparable vaccination rates, such as Brunei (99.95% coverage, 0.0185/100,000) vs. Burkina Faso (100.44%, 1.935/100,000), underscores multifactorial influences including viral strain variations, surveillance capacity gaps, and infrastructure limitations. These disparities highlight the complex interplay between biological, systemic, and socioeconomic determinants of vaccine effectiveness.

Achieving durable diphtheria control requires optimizing both coverage levels and programmatic implementation. Time-sensitive multidose schedules necessitate precise vaccination windows (52) to maximize individual protection, while robust routine immunization systems prove essential for sustaining population-level immunity. Supplementary Table 3 provides complete country-level data, including outlier analyses and threshold sensitivity testing. This comprehensive assessment underscores the urgent need for context-specific strategies addressing both biological thresholds and health system realities.

## 4 Discussion

This study presents a systematic and comprehensive assessment of the global burden of diphtheria from 1990 to 2021, revealing significant reductions in incidence, deaths, and disability-adjusted life years (DALYs). These achievements underscore the historical contributions of the World Health Organization’s Expanded Programme on Immunization (EPI) (53). This progress is attributed to advancements in global healthcare, including the widespread and precise use of antibiotics, improved vaccination coverage, and strengthening of health systems. For the first time, this study documents a negative correlation between global childhood DTP3 vaccination coverage and diphtheria incidence from 1990 to 2021 (*r* = −0.191, *p* = 0.011), validating the central role of immunization programs. However, the presence of a significant threshold effect underscores the urgency of restoring vaccination rates in the post-COVID-19 pandemic era.

Diphtheria, an acute respiratory infection caused by *Corynebacterium diphtheriae*, has a historical trajectory dating back to ancient Greece (54). Until the mid-20th century, it remained a leading cause of childhood mortality globally (55). Transmitted via respiratory droplets, the pathogen forms characteristic pseudomembranes in the pharynx, and its exotoxin can lead to severe complications such as myocarditis and peripheral neuritis (1). Notable historical outbreaks, such as the 1,613 pandemic in Spain (termed the “Year of the Stranglers”), exemplify its lethality. While the introduction of diphtheria toxoid vaccine in 1923 led to a dramatic decline in incidence, with high socio-demographic index (SDI) regions (e.g., Australasia) approaching elimination (ASIR ≤ 0.01 per 100,000), the implications of GBD data necessitate critical reflection. Although current GBD metrics demonstrate substantial improvements over three decades, questions remain: Are these the optimal outcomes of existing prevention strategies? Can we further reduce the disease burden? The risk of diphtheria reemergence, particularly amid post-COVID-19 vaccination declines (56), warrants reevaluation.

Drawing on GBD 2021 data, this study documents 13,000 new diphtheria cases globally in 2021, with 62% of total DALYs attributed to sub-Saharan Africa (Figure 2). This stark regional disparity highlights a profound inequity in health outcomes—low SDI regions exhibit a DALY rate of 26.1 per 100,000, over 100 times that of high SDI regions (Figure 3). For the first time, this research quantifies the nonlinear relationship between the socio-demographic index (SDI) and diphtheria burden (*r* = −0.191, *p* = 0.011). Burden reduction stagnates near zero when SDI ≥ 0.7, while countries with SDI ≤ 0.5 (e.g., Nigeria, SDI = 0.50) report a DALY rate of 46.0 per 100,000 (Figure 3). This finding aligns with health ecology theory (57), indicating that socioeconomic development fosters integrated protective effects through improved sanitation, education, and vaccine accessibility.

As a prototype vaccine-preventable disease (VPD) (58), diphtheria control is highly dependent on vaccination coverage. The 2020 World Health Assembly’s Immunization Agenda 2030 (IA2030) aims to reduce the number of unvaccinated children for the first dose of diphtheria-tetanus-pertussis-containing vaccine (DTPcv1) by 50% and achieve 90% coverage for three doses (DTP3) (59). This study confirms a 0.03 per 100,000 reduction in incidence for each 1% increase in DTP3 coverage (*p* < 0.001), with a threshold effect likely due to herd immunity (threshold ~80%). Incidence reduction plateaus at coverage > 85% (Figure 6), consistent with herd immunity theory. Regional heterogeneity is pronounced: low SDI regions (e.g., Central African Republic) report a DALY rate of 25.3 per 100,000, while high SDI regions (e.g., Norway) near elimination (Figure 3). This disparity reflects the decisive impact of socioeconomic factors on disease control. Notably, despite a 5% decline in coverage during the pandemic (from 86 to 81%) while incidence continued to decrease.

The paradoxical decline in diphtheria incidence during the COVID-19 pandemic can be attributed to three key mechanisms: First, widespread implementation of non-pharmaceutical interventions (NPIs) such as mask mandates and social distancing significantly reduced respiratory pathogen transmission (60). Though the decline in vaccination rates, some improvement in public health awareness, including enhanced hand hygiene and environmental disinfection measures, has effectively reduced the risk of contact transmission for diphtheria. Second, accumulated herd immunity from pre-pandemic vaccination programs provided temporary protection despite coverage interruptions. Third, substantial underreporting occurred due to disrupted disease surveillance systems. However, this apparent decline masks significant long-term risks, as NPI relaxation combined with sustained low vaccination rates may create dangerous immunity gaps. Epidemiological models indicate that maintaining coverage below 80% risks breaching critical herd immunity thresholds, with projections suggesting sub-Saharan Africa could face resurgence rates of 0.5/100,000 by 2026.

Conflict-related barriers to vaccination create complex challenges in fragile settings, as exemplified by multiple case studies: In Ethiopia’s North Wollo region, 6 months of armed conflict destroyed health infrastructure, significantly increasing vaccination dropout rates among children under two (61); in the Central African Republic, vaccine diversion by armed groups (62) has hindered the conversion of international aid into actual coverage; and in northeastern Nigeria, conflict zones showed 3.2-fold higher diphtheria incidence than peaceful areas due to disrupted immunization access (63). These settings also face severe underreporting due to collapsed surveillance systems, masking the true disease burden and distorting regional comparisons.

The 2022–2023 Nigerian diphtheria outbreak demonstrated these systemic failures, with coverage dropping from 75 to 68% amid healthcare disruptions (64). Similarly, Haiti’s heavy reliance on external vaccine supplies—without local production capacity—left its immunization system vulnerable despite international aid (65). These cases reveal two distinct but critical pathways to resurgence: (1) conflict-driven health system collapse and (2) unsustainable aid dependence. To mitigate future outbreaks, tailored strategies must address both scenarios—implementing conflict-resilient delivery systems in unstable regions while strengthening local vaccine production in aid-dependent nations.

Eastern Asia has demonstrated remarkable success in diphtheria control, as evidenced by the region’s steepest decline in DALYs (AAPC: −12.21%; Table 1). This achievement stems from well-designed, sustainable immunization strategies: China’s inclusion of diphtheria vaccine in its national immunization program was supported by stable financial investments (3–5% of health budget), enabling the establishment of a three-tier cold chain monitoring system. China’s “categorized, stratified, and zoned” management model ensured sustainable resource allocation. Similarly, South Korea achieved high coverage by integrating vaccination services with primary healthcare facilities, while Thailand’s multisectoral approach—involving non-health ministries in NCD prevention (66) proved particularly effective in humid subtropical regions where functional homogeneity of healthcare delivery was crucial. These experiences highlight that even with intensified vaccination efforts and external aid, long-term success depends on context-adapted, institutionally-grounded implementation frameworks.

Crucially, all interventions must prioritize achieving and sustaining coverage above 95%, the evidence-based threshold for robust herd immunity[WHO2023] (67). Vaccine coverage greater than the immunization threshold is effective in controlling infectious diseases, and it is critical to meet the thresholds in both low and high SDI countries. Beyond coverage statistics, the quality of vaccination programs warrants thorough evaluation, particularly regarding: (1) cold chain equipment (vaccine refrigerators and freezers), (2) vaccine supplies and consumables, and (3) vaccinator training programs. Through coordinated catch-up campaigns and strengthened surveillance networks, the establishment of dedicated immunization centers has been demonstrated as an effective strategy to enhance service delivery (68).

Three consecutive years of declining vaccination rates (2019–2021) exacerbate the potential for large-scale resurgence (52). With 2021 coverage at 81%, br00eaching the modeled threshold (86.5%) (Figure 7) raises concerns about reintroduction even in high SDI regions. The COVID-19 pandemic severely disrupted routine immunization programs globally, with approximately 25 million children missing essential vaccinations in 2021 alone—60% of whom were concentrated in just 10 low-and middle-income countries (69). WHO African Region data reveal a concerning trend: while DTP3 coverage showed modest recovery in 2022 compared to 2021 levels, it remained below pre-pandemic (2019) benchmarks, accompanied by a growing population of under-or unvaccinated children (70). This pattern, consistently observed across multiple studies, creates substantial immunity gaps that significantly elevate the risk of diphtheria resurgence in vulnerable populations.

The COVID-19 pandemic’s disruption to immunization programs exhibited stark socioeconomic gradients: high-SDI regions leveraged existing advantages—including vaccine stockpiles, adaptable delivery systems, and robust infrastructure—to limit coverage declines and maintain cold chain integrity (69), while low-SDI areas suffered profound setbacks due to aid disruptions, workforce shortages, and systemic vulnerabilities like unstable power grids compromising vaccine stability. This divergence exacerbated health inequities, as high-SDI populations could offset temporary vaccination lapses with alternative protections and baseline health advantages, whereas low-SDI communities—already disproportionately dependent on immunization (70)—faced elevated outbreak risks and severe outcomes from eroded herd immunity, widening preexisting disparities in preventable disease burden.

To mitigate the long-term impacts of the pandemic on diphtheria control, high-SDI regions must prioritize three key interventions: (1) enhancing real-time pathogen genomic surveillance to detect emerging diphtheria variants, (2) intensifying vaccine R&D and technological upgrades, and (3) implementing comprehensive adult booster programs to address coverage gaps evident in countries like France and Germany (71, 72). These regions should also strengthen global health leadership by facilitating affordable technology transfer to lower-SDI areas. Middle-SDI settings require rigorous cost-effectiveness analyses to optimize limited resources, focusing on: (a) selecting the most efficient vaccine combinations [e.g., Tritanrix-HB/Hib—the only commercially available vaccine against diphtheria, tetanus, whole-cell pertussis, hepatitis B, and Hib conjugate (66)], and (b) implementing WHO EPI-recommended combined vaccines to reduce logistical costs (73). Low-SDI areas demand integrated strategies combining nutritional interventions with immunization programs, which have demonstrated 28% (*p* = 0.007) and 12% (*p* = 0.05) reductions in underweight and stunting risks, respectively, (74). In extreme vulnerability settings (e.g., Somalia), essential “basic nutrition package (vitamin A/zinc) + core vaccines” should be delivered through conflict-resilient mobile networks prioritizing pregnant women and under-5 children. Crucially, vaccination metrics must be adjusted to “cold chain compliance rate × actual vaccination completion rate” to account for health system disruptions. Across all SDI levels, interventions must be tailored to local health system capacities.

The long-term disability burden of diphtheria in children, often overlooked, emerges as a critical concern (75). Despite over four decades of vaccination, 2021 GBD data reveal 250,000 child DALYs globally, accounting for 80.5% of total burden. While mortality has declined, the proportion of DALYs attributed to years lived with disability (YLDs) has increased, signaling rising chronic sequelae. Children face distinct clinical profiles: infants present with systemic symptoms (fever, lethargy, respiratory distress) and toxin-mediated damage, while preschoolers exhibit localized symptoms [pharyngitis, hoarseness, croup-like cough, kidney injury (76)] with milder systemic effects. Laryngeal obstruction in children, leading to hypoxic brain injury (77) (cognitive impairment, cerebral palsy), underscores the need to prioritize both survival and quality of life in health initiatives.

This study has limitations: ① The GBD framework’s reliance on modeled data may underestimate disease burden in low-SDI regions, while national aggregation in high-income areas obscures early outbreak signals, hindering timely epidemic detection. Larger sample sizes in certain age groups (e.g., 6–11 months) or high regional heterogeneity in the data may lead to wider confidence intervals (CIs). Such broad CIs reflect the inherent statistical uncertainty in the data, requiring cautious interpretation of the effect estimates. ② Ecological correlations between vaccination and incidence do not establish causality, while global analysis demonstrates a negative correlation between DTP3 vaccination coverage and diphtheria incidence (r = −0.191, *p* < 0.05), the relatively weak strength of this association warrants cautious interpretation of its public health implications. ③ Emerging risk factors such as antibiotic resistance were not incorporated into the analysis—2008–2017 isolates show 17.2% penicillin resistance (10.4% multidrug-resistant) (78). ④ The current GBD model assumes uniform virulence across all *Corynebacterium diphtheriae* strains, an oversimplification that fails to account for mortality variations between toxigenic (tox gene-positive) and non-toxigenic strains (15% vs. 5%, respectively) (79). Furthermore, the model neglects antimicrobial resistance (AMR) patterns, which may lead to substantial underestimation of disease burden. Rising AMR rates correlate with increased treatment failure, prolonged illness duration, and higher complication risks—factors that likely result in systematic underestimation of disability-adjusted life years (DALYs) (80). This lack of strain-specific virulence and AMR differentiation in modeling could misguide clinical intervention prioritization, particularly in resource-limited settings where strain surveillance is sparse, possibly overestimating vaccine efficacy.

Future policy interventions must adopt context-specific strategies for key stakeholders. Governments and international agencies should prioritize children and pregnant women in tailored catch-up vaccination campaigns, leveraging community health networks for outreach in low-access settings. In resource-constrained regions, adult booster programs require decentralized delivery models (e.g., mobile clinics) and age-responsive immunization tracking systems to overcome infrastructure limitations and older adult mobility barriers. Surveillance integration with routine primary care—particularly maternal health services—can simultaneously improve case reporting accuracy (currently suboptimal by 40–60% in conflict areas) and vaccine coverage monitoring.

Future research should prioritize four key directions: First, conduct serological surveillance to dynamically assess population immunity levels, using quantitative anti-toxin IgG antibody testing (integrated with mobile labs and digital reporting systems) to precisely identify protection gaps in adults and vulnerable populations (81). Second, establish a global *C. diphtheriae* genomic surveillance network to track toxigenic strain evolution, antimicrobial resistance patterns (e.g., penicillin-macrolide cross-resistant strains), and phage-mediated transmission mechanisms (82), combining molecular epidemiology for outbreak early-warning. Third, implement cluster-randomized trials to evaluate cost-effectiveness of catch-up strategies (e.g., “3 + 1” booster regimens) in conflict zones/migrant populations (83), while improving cold chain coverage (≥90%) and advocating diphtheria control inclusion in WHO’s Global Vaccine Action Plan through COVID-19-style equity mechanisms. Finally, apply mixed-methods (quantitative surveys + qualitative interviews) to systematically analyze vaccination barriers in low-SDI settings, particularly health system fragmentation (e.g., supply chain disruptions) and vaccine hesitancy (e.g., misinformation), while developing community-engaged interventions (mobile clinics, culturally-adapted health education). These priorities will provide evidence-based tools for WHO’s Immunization Agenda 2030, facilitating the transition from “coverage-focused” to “immunity quality-driven” strategies to achieve sustainable diphtheria elimination.

## 5 Conclusion

In conclusion, this study highlights significant progress in reducing global diphtheria burden over three decades, primarily driven by vaccination and healthcare improvements. However, persistent regional disparities, particularly in low-socio-demographic index (SDI) regions, underscore systemic inequities in vaccine access and healthcare resilience. The inverse relationship between SDI and disease burden emphasizes the role of socioeconomic determinants in shaping outcomes, aligning with health ecology principles.

Post-COVID-19 declines in vaccination coverage pose critical resurgence risks, necessitating urgent efforts to restore immunization rates and rebuild herd immunity thresholds. While transient protective effects from pandemic interventions delayed outbreaks, sustained vaccination campaigns are essential to prevent future epidemics. Emerging challenges, including rising pediatric disability and gender-specific incidence, demand tailored strategies addressing biological and social vulnerabilities.

To achieve elimination, integrated approaches are required: equitable vaccine distribution, strengthened healthcare systems in low-SDI regions, and adaptive vaccination strategies. By prioritizing post-pandemic recovery of vaccination programs and aligning with global health frameworks like the WHO’s Immunization Agenda 2030, these efforts can bridge gaps in health equity and accelerate progress toward diphtheria elimination. This research provides a vital evidence base for policymakers and stakeholders, emphasizing the importance of sustained action to secure long-term public health gains.

## Data Availability

The datasets presented in this study can be found in online repositories. The names of the repository/repositories and accession number(s) can be found in the article/Supplementary material.
