# Supplementary material for: Global burden of diphtheria, 1990–2021: a 204-country analysis of socioeconomic inequality based on SDI and DTP3 vaccination differences before and after the COVID-19 pandemic (GBD 2021)
Source: Front Public Health. 2025 Jun 20;13:1597076. doi: 10.3389/fpubh.2025.1597076 (PMC12226553; doi:10.3389/fpubh.2025.1597076)
Supplement: Supplementary file 1 [file Table_1.docx]

**Tables：**

| Location | 1990 | | | 2021 | | AAPC (95% CI) |
| --- | --- | --- | --- | --- | --- | --- |
|  | Number (95%UI) | ASR ( 95% UI) | | Number (95%UI) | ASR ( 95% UI) |  |
| **Incidence** |  |  | |  |  |  |
| Global | 87081.5(65855,118554.6) | | 1.5(1.1,2) | 13312.9(8940.1,18500) | 0.2(0.1,0.3) | -6.29 (-6.80 - -5.77) |
| Male | 41192.4(30412.2,57161) | | 1.3(1,1.8) | 6788.1(4652.6,9900.8) | 0.2(0.1,0.3) | -6.02 (-6.41 - -5.63) |
| Female | 45889.1(33024.2,63354.6) | | 1.6(1.1,2.2) | 6524.8(4135.6,9439.7) | 0.2(0.1,0.3) | -6.48 (-6.98 - -5.98) |
| Low SDI | 52166.3(37256,75244.7) | | 6.5(4.8,9.3) | 11145.3(7169.9,16089.6) | 0.7(0.5,1.1) | -6.78 (-7.04 - -6.52) |
| Low-middle SDI | 25829.5(18679.7,36187.6) | | 1.7(1.2,2.3) | 1455.3(1146.2,1896.6) | 0.1(0.1,0.1) | -9.26 (-10.04 - -8.47) |
| Middle SDI | 7286.8(5918.4,8952.2) | | 0.4(0.3,0.5) | 614.6(519.8,717.1) | 0(0,0) | -8.18 (-8.48 - -7.87) |
| High-middle SDI | 1705.5(1465.4,1999.8) | | 0.2(0.1,0.2) | 55.4(46,68.2) | 0(0,0) | -11.12 (-12.13 - -10.09) |
| High SDI | 50.2(38.6,64.3) | | 0(0,0) | 27.7(21.8,34.6) | 0(0,0) | -2.55 (-3.60 - -1.48) |
| Andean Latin America | 151.4(90.5,245.3) | | 0.3(0.2,0.5) | 5.6(3.8,8) | 0(0,0) | -10.26 (-11.26 - -9.24) |
| Australasia | 0.1(0.1,0.1) | | 0(0,0) | 2.2(1,4.7) | 0(0,0) | 9.72 (4.75 - 14.92) |
| Caribbean | 262.9(127.5,489.9) | | 0.7(0.3,1.2) | 62.9(33.9,108) | 0.2(0.1,0.3) | -4.60 (-4.74 - -4.45) |
| Central Asia | 131.2(85.6,183.3) | | 0.2(0.1,0.2) | 12.5(8.5,16.8) | 0(0,0) | -7.99 (-8.89 - -7.07) |
| Central Europe | 12.7(10,16.2) | | 0(0,0) | 2.1(1.5,2.9) | 0(0,0) | -6.12 (-7.06 - -5.18) |
| Central Latin America | 75.5(61.9,93.4) | | 0(0,0) | 3.1(2,4.6) | 0(0,0) | -10.44 (-11.45 - -9.41) |
| Central Sub-Saharan Africa | 4572.1(2464,7587.6) | | 5.1(2.9,8.1) | 878.3(547,1415.8) | 0.5(0.3,0.8) | -7.08 (-7.32 - -6.83) |
| East Asia | 2922.5(2309.3,3634.6) | | 0.3(0.2,0.3) | 83.6(66.3,105.9) | 0(0,0) | -11.30 (-11.55 - -11.05) |
| Eastern Europe | 948.4(786.6,1141.4) | | 0.4(0.4,0.5) | 4.9(2.4,9.5) | 0(0,0) | -13.51 (-20.28 - -6.16) |
| Eastern Sub-Saharan Africa | 15635.8(10383.8,23362.6) | | 5.1(3.6,7.5) | 2745.1(1759.2,4290.3) | 0.5(0.3,0.8) | -7.15 (-7.32 - -6.98) |
| High-income Asia Pacific | 20.6(13.1,30.4) | | 0(0,0) | 2.9(2.3,3.6) | 0(0,0) | -5.56 (-5.78 - -5.34) |
| High-income North America | 5.5(4.5,6.7) | | 0(0,0) | 15.8(12.4,20.2) | 0(0,0) | 3.02 (2.37 - 3.66) |
| North Africa and Middle East | 5727.9(3481.4,9269.4) | | 1.3(0.8,2) | 330.2(228.8,476.7) | 0.1(0,0.1) | -10.11 (-10.79 - -9.41) |
| Oceania | 132.7(73.3,217.4) | | 1.6(0.9,2.5) | 195.9(101.5,326.1) | 1.1(0.6,1.8) | -0.93 (-1.15 - -0.70) |
| South Asia | 22585.6(16350.5,31978.1) | | 1.6(1.2,2.2) | 719.7(586.9,886.2) | 0(0,0) | -11.11 (-11.37 - -10.86) |
| Southeast Asia | 7224(5411.8,9830.9) | | 1.3(1,1.8) | 595.2(485.5,726.1) | 0.1(0.1,0.1) | -6.91 (-7.68 - -6.13) |
| Southern Latin America | 10(6.6,15.3) | | 0(0,0) | 0.1(0.1,0.2) | 0(0,0) | -12.49 (-22.40 - -1.31) |
| Southern Sub-Saharan Africa | 125.8(96.2,168.1) | | 0.2(0.2,0.3) | 84.6(66.7,108.4) | 0.1(0.1,0.1) | -2.20 (-2.68 - -1.71) |
| Tropical Latin America | 133.7(107.1,167.7) | | 0.1(0.1,0.1) | 5.8(4.4,7.3) | 0(0,0) | -9.95 (-10.86 - -9.04) |
| Western Europe | 2(1.5,2.6) | | 0(0,0) | 2.1(1.7,2.8) | 0(0,0) | -0.31 (-1.38 - 0.78) |
| Western Sub-Saharan Africa | 26401.1(17691.9,39113.7) | | 8(5.4,11.9) | 7560.2(4178.4,11416) | 1(0.6,1.5) | -6.40 (-6.56 - -6.24) |
| **Death** |  | |  |  |  |  |
| Global | 25446.4(19391,34380.6) | | 0.4(0.3,0.6) | 3824.7(2610.3,5393.8) | 0.1(0,0.1) | -6.19 (-6.63 - -5.76) |
| Male | 12980.5(9682.3,18272.2) | | 0.4(0.3,0.6) | 2091.6(1387.5,3064.5) | 0.1(0,0.1) | -6.02 (-6.40 - -5.63) |
| Female | 12465.9(9379,16928.8) | | 0.4(0.3,0.6) | 1733.1(1125.8,2499.9) | 0.1(0,0.1) | -6.38 (-6.89 - -5.88) |
| Low SDI | 16394(11982,23244.3) | | 2(1.5,2.8) | 3335.4(2197.9,4834.5) | 0.2(0.1,0.3) | -6.89 (-7.18 - -6.59) |
| Low-middle SDI | 7630.2(5654.8,10215.5) | | 0.5(0.4,0.6) | 381.7(295.5,497.7) | 0(0,0) | -9.56 (-10.29 - -8.83) |
| Middle SDI | 1242.9(1051.4,1476.1) | | 0.1(0.1,0.1) | 93.9(80.8,106.7) | 0(0,0) | -8.31 (-8.62 - -8.01) |
| High-middle SDI | 159.7(139.2,188.2) | | 0(0,0) | 6.7(5.8,8) | 0(0,0) | -9.74 (-12.96 - -6.41) |
| High SDI | 8.9(6.8,11.4) | | 0(0,0) | 4.2(3.5,5) | 0(0,0) | -2.81 (-4.80 - -0.78) |
| Andean Latin America | 58.4(34.7,94.4) | | 0.1(0.1,0.2) | 1.8(1.2,2.6) | 0(0,0) | -10.73 (-11.97 - -9.48) |
| Australasia | 0(0,0) | | 0(0,0) | 0.3(0.2,0.5) | 0(0,0) | 13.43 (-0.57 - 29.39) |
| Caribbean | 104.6(51,185.4) | | 0.3(0.1,0.5) | 22.9(11.5,39.4) | 0.1(0,0.1) | -4.81 (-5.72 - -3.89) |
| Central Asia | 19.1(12.5,27.8) | | 0(0,0) | 1.6(1,2.2) | 0(0,0) | -7.67 (-11.13 - -4.09) |
| Central Europe | 1.1(0.9,1.4) | | 0(0,0) | 0.2(0.1,0.2) | 0(0,0) | -6.69 (-8.62 - -4.72) |
| Central Latin America | 25.9(21.9,30.9) | | 0(0,0) | 0.9(0.6,1.3) | 0(0,0) | -10.99 (-12.89 - -9.04) |
| Central Sub-Saharan Africa | 1435.7(755,2451.2) | | 1.5(0.8,2.6) | 246.6(150,400.5) | 0.1(0.1,0.2) | -7.38 (-7.74 - -7.01) |
| East Asia | 373.4(309.8,460.4) | | 0(0,0) | 9.2(7.6,11.3) | 0(0,0) | -11.74 (-12.20 - -11.28) |
| Eastern Europe | 49.7(43.7,55.8) | | 0(0,0) | 0.3(0.1,0.7) | 0(0,0) | -11.01 (-19.89 - -1.15) |
| Eastern Sub-Saharan Africa | 4952(3309,7265.8) | | 1.6(1.1,2.3) | 785.6(499.6,1230.3) | 0.1(0.1,0.2) | -7.46 (-8.00 - -6.91) |
| High-income Asia Pacific | 3.6(2.4,5.2) | | 0(0,0) | 0.5(0.4,0.6) | 0(0,0) | -5.75 (-7.77 - -3.68) |
| High-income North America | 0.9(0.8,1.1) | | 0(0,0) | 2.5(2,3) | 0(0,0) | 3.01 (-1.23 - 7.43) |
| North Africa and Middle East | 1250.1(772.2,2007.2) | | 0.3(0.2,0.4) | 65.8(44.9,90.7) | 0(0,0) | -10.08 (-11.28 - -8.86) |
| Oceania | 17.2(9.6,28.8) | | 0.2(0.1,0.3) | 25.2(12.7,43.7) | 0.1(0.1,0.2) | -0.94 (-1.47 - -0.40) |
| South Asia | 7623.6(5595.7,10237.5) | | 0.5(0.4,0.7) | 217.6(178.5,265.4) | 0(0,0) | -11.36 (-11.82 - -10.90) |
| Southeast Asia | 1006(762.8,1337.4) | | 0.2(0.1,0.2) | 68.1(58.1,80.2) | 0(0,0) | -7.29 (-8.10 - -6.46) |
| Southern Latin America | 2.2(1.5,3.1) | | 0(0,0) | 0(0,0) | 0(0,0) | -17.13 (-26.53 - -6.54) |
| Southern Sub-Saharan Africa | 33.8(26,43.5) | | 0.1(0,0.1) | 20.7(16.6,26.1) | 0(0,0) | -2.27 (-3.06 - -1.46) |
| Tropical Latin America | 52.4(43.4,61.9) | | 0(0,0) | 2(1.5,2.5) | 0(0,0) | -9.76 (-11.76 - -7.71) |
| Western Europe | 0.4(0.3,0.4) | | 0(0,0) | 0.3(0.3,0.4) | 0(0,0) | 1.11 (-2.23 - 4.58) |
| Western Sub-Saharan Africa | 8436.4(5858.6,12738.7) | | 2.5(1.8,3.8) | 2352.7(1400.8,3526.4) | 0.3(0.2,0.5) | -6.50 (-6.82 - -6.18) |
| **DALYs** |  | |  |  |  |  |
| Global | 2148470.2(1629731.5,2922276.7) | | 35.3(26.8,47.9) | 318203.8(213604.1,453613.5) | 4.8(3.2,6.8) | -6.18 (-6.63 - -5.74) |
| Male | 1091352(805877.3,1545749.8) | | 34.8(25.8,49.3) | 173027.1(113250,256175.6) | 5(3.3,7.5) | -6.01 (-6.39 - -5.62) |
| Female | 1057118.2(789929.1,1446305.8) | | 35.7(26.7,48.7) | 145176.7(92464.7,211381.7) | 4.5(2.9,6.6) | -6.31 (-6.72 - -5.90) |
| Low SDI | 1399997(1017859.6,1993074.7) | | 164.8(120.7,232.6) | 281252.9(183171.7,411215.8) | 17.7(11.7,25.7) | -6.92 (-7.22 - -6.61) |
| Low-middle SDI | 634560.5(466846.3,857975.3) | | 38.8(28.7,51.9) | 29543.5(22294.3,39256.2) | 1.5(1.1,2) | -9.69 (-10.41 - -8.96) |
| Middle SDI | 100624.6(84740.7,120479) | | 5.2(4.3,6.1) | 6523.7(5514.8,7574.7) | 0.3(0.3,0.4) | -8.51 (-8.79 - -8.23) |
| High-middle SDI | 11756.9(10075.9,14207.2) | | 1.2(1.1,1.5) | 406.8(345.6,492.4) | 0(0,0.1) | -9.95 (-12.65 - -7.18) |
| High SDI | 639.3(472.2,856.6) | | 0.1(0.1,0.1) | 253.7(212.6,303) | 0(0,0) | -3.17 (-4.81 - -1.50) |
| Andean Latin America | 4920.8(2857.6,8063.8) | | 9.6(5.7,15.6) | 133.6(87.3,190.1) | 0.2(0.1,0.3) | -11.03 (-12.29 - -9.76) |
| Australasia | 0.9(0.7,1.2) | | 0(0,0) | 17.1(8.9,33.1) | 0.1(0,0.1) | 13.06 (-1.44 - 29.68) |
| Caribbean | 8864.6(4236.6,15902.9) | | 21.8(10.4,39.3) | 1836.2(881,3231.4) | 4.6(2.2,8.2) | -4.89 (-5.80 - -3.97) |
| Central Asia | 1571.4(980.2,2334) | | 1.8(1.2,2.6) | 118(74.5,171.7) | 0.1(0.1,0.2) | -7.89 (-10.81 - -4.87) |
| Central Europe | 73.7(57.8,95.5) | | 0.1(0.1,0.1) | 8.2(6.4,10.6) | 0(0,0) | -8.08 (-9.74 - -6.39) |
| Central Latin America | 2084.2(1764.4,2510.8) | | 1(0.8,1.2) | 59.5(39.7,88.2) | 0(0,0) | -11.39 (-13.29 - -9.44) |
| Central Sub-Saharan Africa | 122683.3(64097,210944) | | 127.2(67.4,217) | 19730.4(11792.3,32839.9) | 10.4(6.4,16.8) | -7.67 (-8.05 - -7.29) |
| East Asia | 29741.4(24386.3,37160.1) | | 2.6(2.1,3.2) | 525.6(432,632.6) | 0(0,0.1) | -12.21 (-12.67 - -11.74) |
| Eastern Europe | 3095.6(2700.5,3477.3) | | 1.6(1.4,1.8) | 16.4(7.7,32.6) | 0(0,0) | -12.16 (-21.00 - -2.33) |
| Eastern Sub-Saharan Africa | 420964.3(278434.1,622149.4) | | 127.1(85.4,185.4) | 64054.1(39783.4,102408.1) | 10.9(6.9,17.1) | -7.46 (-7.92 - -7.00) |
| High-income Asia Pacific | 236.3(149.5,348.7) | | 0.2(0.1,0.3) | 29.8(24.4,36.9) | 0(0,0) | -5.72 (-7.80 - -3.60) |
| High-income North America | 66.4(57.8,76.9) | | 0(0,0) | 152.7(125.1,183.4) | 0.1(0,0.1) | 2.79 (-1.45 - 7.22) |
| North Africa and Middle East | 104120.4(63041.7,170028.6) | | 21.6(13.4,34.7) | 5078.2(3383.1,7322.5) | 0.8(0.5,1.2) | -10.14 (-11.33 - -8.94) |
| Oceania | 1401.9(741.9,2405.8) | | 15.2(8.5,25.3) | 2037.9(970.8,3640.7) | 11.1(5.5,19.4) | -0.89 (-1.43 - -0.35) |
| South Asia | 629133.1(455840.1,850080.8) | | 42.2(31,56.9) | 15977.5(12739.7,19825.5) | 0.9(0.7,1.2) | -11.56 (-12.04 - -11.09) |
| Southeast Asia | 82358.3(61670.7,111098.3) | | 14.6(11,19.5) | 4734.3(3905.9,5709.9) | 0.8(0.6,0.9) | -7.49 (-8.35 - -6.63) |
| Southern Latin America | 178.4(122.4,253.2) | | 0.3(0.2,0.5) | 1.3(0.9,1.7) | 0(0,0) | -17.62 (-27.04 - -6.98) |
| Southern Sub-Saharan Africa | 2698.7(2005.8,3534.2) | | 4(3,5.1) | 1522.8(1199.5,1973.7) | 1.9(1.5,2.4) | -2.38 (-3.19 - -1.55) |
| Tropical Latin America | 4490.2(3699.1,5313.3) | | 2.6(2.2,3.1) | 159(119.8,204.7) | 0.1(0.1,0.1) | -9.89 (-11.94 - -7.79) |
| Western Europe | 25.4(20,30.8) | | 0(0,0) | 21.7(18,26.6) | 0(0,0) | 1.07 (-2.33 - 4.59) |
| Western Sub-Saharan Africa | 729760.9(506063.3,1103446.4) | | 215.1(148.8,324.2) | 201989.6(119566.2,303772.7) | 26.1(15.6,39.2) | -6.53 (-6.85 - -6.21) |

**Table 1.** **Incidence, death, and DALYs of Diphtheria in 1990 and 2021, and Trends Over Time**
